# Administrative Perspectives on Digital Workflow Transformation and Artificial Intelligence Implementation in Dental Clinics

**DOI:** 10.3390/dj14040206

**Published:** 2026-04-02

**Authors:** Alin Flavius Cozmescu, Ana Cernega, Andreea Cristiana Didilescu, Marina Meleșcanu Imre, Bogdan Dimitriu, Silviu-Mirel Pițuru

**Affiliations:** 1 Department of Organization, Professional Legislation and Management of the Dental Office, Faculty of Dental Medicine, “Carol Davila” University of Medicine and Pharmacy, 17-23 Plevnei Street, 020021 Bucharest, Romania; alin-flavius.cozmescu@drd.umfcd.ro (A.F.C.); silviu.pituru@umfcd.ro (S.-M.P.); 2 Department of Embryology and Microbiology, Faculty of Dental Medicine, “Carol Davila” University of Medicine and Pharmacy, 010221 Bucharest, Romania; andreea.didilescu@umfcd.ro; 3 Department of Prosthodontics, Faculty of Dental Medicine, “Carol Davila” University of Medicine and Pharmacy, 17-23 Calea Plevnei, 010221 Bucharest, Romania; marina.imre@umfcd.ro; 4 Department of Endodontics, Faculty of Dental Medicine, “Carol Davila” University of Medicine and Pharmacy, 17-23 Plevnei Street, 020021 Bucharest, Romania; bogdan.dimitriu@umfcd.ro

**Keywords:** artificial intelligence, digital workflow, dental clinic management, healthcare digitalization, efficiency, organizational change

## Abstract

**Background/Objectives**: The digital transformation of dental practice is positioning artificial intelligence (AI) as a key tool for both clinical support and administrative optimization. While clinical uses of AI are well documented, there is limited evidence on managerial perspectives. This study explored how dental clinic managers view digital workflow transformation and AI implementation. **Methods**: A cross-sectional questionnaire-based study was conducted among 200 managers of dental clinics from urban and rural areas in Bucharest, Romania. The survey evaluated perceived difficulty and availability related to digitalization, current use of digital tools, demographic characteristics (age, professional experience, practice environment), and attitudinal dimensions reflecting digital pragmatism and efficiency versus human impac. **Results**: Managers demonstrated moderate digital pragmatism (median 2.84, IQR 2.29–3.44), embracing AI mainly when linked to efficiency, operational control, and economic sustainability. Lower perceived difficulty was associated with higher availability, current use of digital tools, younger age, and fewer years of managerial experience. Urban managers were more likely than rural managers to report higher availability and current use of digital tools, although this comparison should be interpreted cautiously given the small rural subgroup. Efficiency considerations outweighed human-impact concerns (median 3.9, IQR 3.46–4.2), yet caution persisted toward solutions requiring major organizational restructuring or potentially affecting clinician–patient interaction. This study is a pilot, exploratory investigation aimed at generating preliminary insights into the phenomenon of interest and refining the methodological approach and hypotheses for subsequent, larger-scale research. **Conclusions**: Dental clinic managers approach AI adoption through an efficiency-driven and risk-aware framework, favoring incremental digital integration over disruptive transformation. The results underline the need for context-sensitive implementation strategies, managerial training, and targeted support, to ensure that AI-enhanced workflows improve efficiency while preserving organizational stability and patient-centered care.

## 1. Introduction

Artificial intelligence was originally conceived as a demonstration of competence and technological progress. The founding milestones remain clear: in 1950, Alan Turing proposed the famous test that bears his name to imagine when a machine could generate responses indistinguishable from those of a human mind, and in 1956, at Dartmouth, John McCarthy officially legitimized the term “artificial intelligence” launching a new field of research. For many decades, dreams were beyond the technical possibilities, but with the growth of computing power, the massive digitization of clinical data and the refinement of machine learning models, artificial intelligence has moved beyond complex mathematical calculations and into the deep workings of the medical system [1,2].

Today, deep learning algorithms are scanning entire sections of radiologic imaging [3,4] or histopathologic tissue at a pace inaccessible to the medical specialist, identifying micro-anomalies that herald early-stage malignancies [5] or calculating the likelihood of decompensation of a heart failure patient based on an impressive array of physiologic, demographic and genetic variables [6]. The paradigm has shifted substantially in recent years, so we are no longer just talking about establishing a diagnosis assisted by computer-assisted information, but augmented medicine, where artificial intelligence becomes a partner in clinical deliberation [7,8]. The impact, however, goes beyond the therapeutic frontier. In the clinical space, artificial intelligence is rethinking resource allocation, estimating emergency flows, calibrating point-of-care assignments and optimizing the operative calendar [9,10,11,12]; in pharmacology, generative algorithms are exploring the molecular space, reducing the duration of preclinical phases [13,14,15,16]. If we talk about implementing artificial intelligence in everyday patient life, predictive models detect subtle deviations from the usual baseline parameters, data transmitted by wearable smart devices, alerting the medical team even before the patient notices the first symptoms [17,18].

In particular, virtual assistants based on AI technologies take over some of the initial triage, guide the patient through the system and can improve access to care in regions with a shortage of specialists [19,20].

This transformation does not come without safety and ethical costs. Models have the potential to amplify bias in historical datasets, and their opacity raises questions about the explainability of automated decisions and the distribution of responsibility between developer, clinician and institution [21]. In the European context, the (EU) AI Regulation together with already well-established rules on personal data protection (GDPR) require a governance framework that focuses on transparency, verification and security [22,23]. At the same time, public health policies also need to prevent the emergence of a discriminatory medical practice where only centers with state-of-the-art digital infrastructure in big cities benefit from the technological advantages of algorithms [24,25,26,27].

In essence, the penetration of AI into the healthcare system signals a shift from reactive medicine, oriented towards episodic disease resolution, to predictive, preventive and personalized medicine. For this metamorphosis to reach its full potential, it will require an alliance between traditional clinical competence and emerging digital intelligence, underpinned by an ethical and regulatory architecture capable of protecting patient rights, ensuring equity of access, and transforming data richness into a genuine public health gain [24,25].

The administrative component and the role of the practice manager are essential for the cohesion of the workflow in dentistry, where clinical practice depends on the fine synchronization between planning, human resources, and medical infrastructure [28]. The manager orchestrates recruitment, integration, and skills development, assigning tasks according to the team profile (doctors, assistants, front office) and demand variability. A skill-mix and task shifting approach (e.g., delegating prophylaxis or health education to newly graduated doctors, follow-ups to reception) allows the specialist to focus their time on clinical decisions and the organization to stabilize the cost per procedure and the quality of services offered [29,30].

Digitization and artificial intelligence improve these capabilities: smart slot scheduling, adaptive reminders, rule-guided initial triage, procedural duration estimation, decision support for treatment plans, automatic billing, and inventory management. However, value only emerges when the manager designs interoperable processes, establishes data governance (informed consent, GDPR compliance), and manages change: mapping flows, training digital skills, and defining Key Performance Indicators (waiting time, chair utilization, no-show rates, costs) [31,32].

In parallel with process digitization, the “human factor” remains a decisive variable in how AI is perceived, adopted, and sustained in dental clinics. A useful introductory framework for structuring this dimension is the DISC behavioral-style model, which offers a shared vocabulary for describing differences in communication preferences, decision pace, risk tolerance, and response to change. In studies of technology implementation, such individual and team-level differences can shape readiness, resistance, and the practical uptake of new digital tools. In the context of dental practice management, DISC can therefore support a more coherent discussion of how managers calibrate team dynamics, allocate responsibilities, and design communication strategies when AI becomes increasingly embedded in daily workflows. By framing workforce variability in an operational (rather than clinical) manner, DISC can help connect HR management choices with safer, more predictable AI integration [33].

The aim of the proposed research is to explore dental clinic managers’ perceptions of the contribution of AI and digitization to efficiency, quality, and patient experience, as well as barriers to implementation. The implications for the entire team: for doctors, more clinical time and caring communication, supported by explainable algorithms; for patients, fast access, transparency, and personalization with guaranteed data protection; for management and administration, the need for organizational redesign, advanced digital skills, and continuous monitoring of performance and risks [34,35,36].

The question proposed in the research is to see “*From an administrative perspective, how, and through what mechanisms, does the integration of AI and digital technologies transform workflow design, resource allocation, and decision-making in dental clinics?*”.

## 2. Materials and Methods

The Materials and Methods section specifies the study architecture, research design, participant eligibility and recruitment, measurement instruments, procedural sequence, and analytical strategy. To ensure methodological transparency and rigor, reporting adhered to the STROBE checklist for cross-sectional investigations. We implemented a quantitative, cross-sectional survey targeting dental managers, aimed at characterizing their perceptions, attitudes, and availability toward integrating digitalization and artificial intelligence into the administrative and operational domains of dental practice (e.g., workflow coordination, staffing and resource planning, patient communication, and data governance/compliance). For the purposes of this study, the digital dentistry component was operationalized across five workflow-related subdomains: appointment/scheduling, diagnosis, treatment planning, patient feedback, and follow-up/recall. These domains were selected to reflect common managerial and clinical interfaces where digital tools and AI-supported applications may be integrated into routine dental care. In this study, “perceived difficulty” denotes the respondent’s perceived effort/complexity of implementing AI in routine workflows, while “availability” refers to the perceived presence and accessibility of AI solutions and enabling resources within the organization (technical infrastructure, financial capacity, and operational support). This manager-centric framing captures organization-level dynamics of technology adoption, complementing clinical perspectives and enabling inference about how digital tools and AI reshape decision-making and practice management.

Statistical analysis was performed using IBM SPSS Statistics for Windows, version 25 (IBM Corp., Armonk, NY, USA) and Microsoft Office Excel/Word 2024. Quantitative variables were reported as means with standard deviations or medians with interpercentile ranges; their distribution was assessed using the Shapiro–Wilk test. Non-parametric independent quantitative variables were compared between investigated factors via Kruskal–Wallis H tests (with Dunn–Bonferroni post-hoc tests), while correlations between non-parametric independent quantitative variables were evaluated using Spearman’s rho correlation coefficient. Qualitative variables were expressed as absolute values or percentages, and intergroup differences were tested with Fisher’s Exact Test. Z-tests with Bonferroni corrections provided further detail in contingency tables.

The investigated subdomains were as follows: scheduling, diagnosis, treatment planning, feedback, and follow-up (dispensarization), each containing 15 items in the survey. In each of the subdomains, exploratory Principal Component Analysis (PCA) models were used, retaining the first component with the highest explained variance (largest eigenvalue, λ), and with items included in the first component having at least an absolute loading of ≥0.30. Analyzing all items in the survey, an overall exploratory PCA was made in order to observe overall digitalization scores, in which four components emerged.

Each item received a score from 1 (minimum interest) to 5 (maximum interest), reversed for items showing negative PCA coefficients. The overall score was the arithmetic mean of all included items. PCA was used only for the exploration of item loading patterns, in which items that needed reverse scoring could be identified; as such, validation assessments for PCA models were not necessary.

Observed scores in the exploratory PCA models were validated via Cronbach’s alpha, where internal consistency was measured, examining changes in the coefficient upon item removal. Statistical significance was set at α = 0.05.

The main eligibility criteria were managers actively involved in the operational and/or strategic management of dental practices in Bucharest and surrounding areas who were willing to participate and complete the questionnaire. To be included, participants had to hold one of the following organizational positions: practice manager/office manager, clinic administrator, operations manager, medical director with managerial responsibilities, or owner-manager. There were no restrictions on age, gender, or length of service in a managerial role. Individuals who did not have current decision-making responsibilities in areas such as workflow planning, human resource allocation, budgeting, or digitization were excluded.

Participants were recruited using a purposive/convenience sampling approach. The questionnaire was disseminated through direct communication and professional channels, including online platforms frequently accessed by dental clinic managers and owners in Romania. Recruitment targeted managers actively involved in operational and/or strategic decision-making in dental practices located in Bucharest and surrounding areas. Given the pragmatic dissemination strategy, the study did not rely on a closed sampling frame, and a precise response rate could not be calculated.

The study population included professionals in dental service management. Independent variables included demographic and professional attributes: age, gender, practice setting, and level of management experience. This framework sought to provide a subtle understanding of the factors influencing the adoption of AI and digital technologies in dental management.

Informed consent was obtained prior to data collection, and responses were aggregated and analyzed according to ethical and academic standards. The questionnaire was reviewed and approved by the Scientific Research Ethics Committee of the Carol Davila University of Medicine and Pharmacy in Bucharest (code PO-35-F-03, no. 28.287 of 1 October 2024).

The study was conducted between October 2024 and March 2025. There were no missing data, as all questionnaires were completed in full.

No formal sample size calculation was performed, as the research was designed as a pilot study focused on the integration of AI and digitization in practice management and the implications for resource allocation and, indirectly, for the doctor-patient relationship. Limitations include the uneven distribution of managerial categories by age (more frequent responses among younger participants), experience, and the time frame for data collection. These issues can be addressed in future studies; the present research serves as a starting point and guide.

## 3. Results

### 3.1. Demographic Results

A total of 200 dental practice managers completed the questionnaire, providing an overview of managerial perspectives on digitalization and AI integration in clinic workflows, including perceived effects on patient communication and experience, as well as concerns related to data security and governance, implementation/maintenance costs, and regulatory and legal implications. Participants were recruited from dental clinics and practices in Bucharest and the surrounding areas, including nearby rural communities, capturing a broad range of organizational profiles and service structures.

However, these findings should be interpreted with caution, as the number of respondents from rural areas was limited to only 8 participants, which represents an important study limitation; therefore, the observed differences should be regarded primarily as exploratory and as a useful direction for future research based on larger and more balanced samples.

The sample was predominantly urban-based (96%) with 192 participants (96.0%) practicing in urban settings and 8 participants (4.0%) in rural settings, and included 58.6% women and 41.4% men. Age distribution showed a clear concentration in the mid-career strata: 25–30 years (11.5%), 31–35 (22.5%), 36–40 (34%), 41–45 (19.5%), 46–50 (10.5%), 51–55 (1.5%), and >60 (0.5%). Professional experience followed a similar pattern of consolidation-phase predominance, with most respondents reporting 4–10 years (49%), followed by 11–15 years (19.5%), 0–3 years (14%), 16–20 years (11.5%), 21–25 years (3.5%), and 26–30 years (2.5%). Overall, the demographic structure indicates a cohort largely positioned in the active development and stabilization phase of managerial practice, supported by a substantial experienced core, which is relevant for interpreting attitudes toward AI-driven workflow transformation.

### 3.2. Subdomains of Dental Treatment

Stages such as scheduling, diagnosis, treatment planning, administration/dispensing, and feedback collection are at the forefront of current and future digital tools. From an administrative and organizational perspective, maintaining the doctor-patient relationship, increasing efficiency, data interoperability, and concerns about data security and protection were central elements in the responses of dental practice managers throughout the questionnaire.

**Figure 1:**
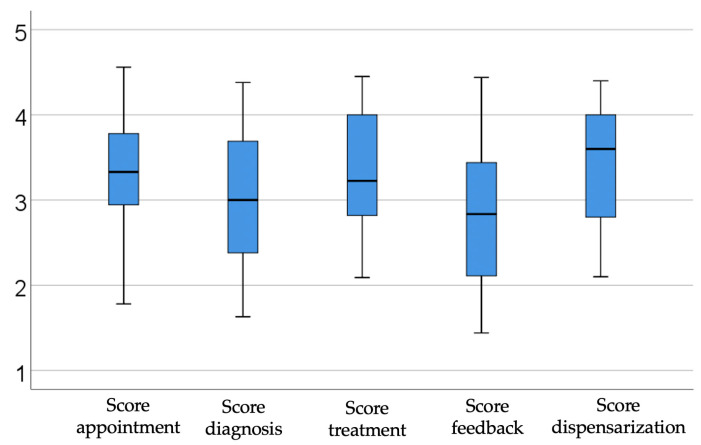
Illustrating scores by subdomains.

Figure 1 shows the description of interest scores by subdomain. The results show that the highest interest scores were for scheduling (median = 3.33, IQR = 2.91–3.78) and dispensing (median = 3.6, IQR = 2.8–4), where the level of interest was medium to high, while the lowest interest score was for feedback (median = 2.83, IQR = 2.11–3.44), where the level of interest was medium to low. Considering that the items focused on the perception of artificial intelligence implementation and process digitization in dental practices, the median scores indicate the highest interest in areas with immediate operational impact: dispensing and scheduling. Distribution of the managers according to the answers for each item in the survey is detailed in the Supplementary Material in Table S1. Construction of scores in PCA is detailed in the Supplementary Material in Table S2.

These findings may reflect increased concern about the medico-legal risks that tend to arise most frequently during the pre-treatment and post-treatment phases. At the same time, these two stages have a particularly strong impact on the clinician–patient relationship and on overall patient satisfaction, which in turn can influence patient referrals and future care experiences. The results regarding the treatment stage suggest openness to decision support tools, but still with some reticence.

In contrast, the diagnosis and feedback stages have the lowest medians and the highest dispersion, indicating caution and heterogeneity among managers. Overall, managers appeared to view the implementation of artificial intelligence in dental practices primarily in relation to administrative efficiency and continuity of care (appointments, reminders, recalls), while remaining cautious where technologies directly interfere with clinical practice or communication.

### 3.3. The Perceived Degree of Difficulty

#### 3.3.1. Analysis of the Perception of the Degree of Difficulty in Relation to the Professional Environment

**Figure 2:**
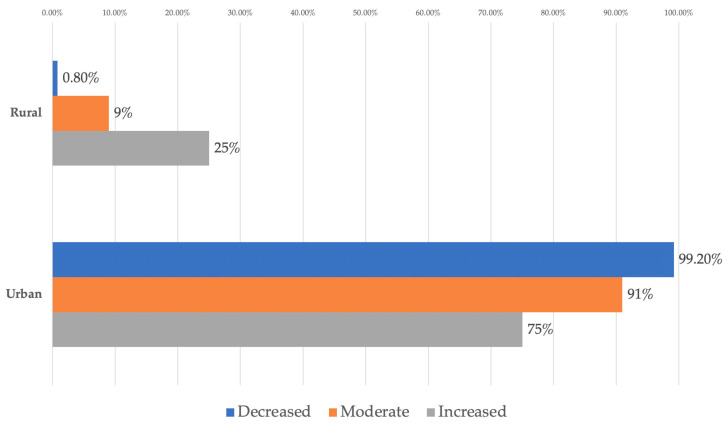
Distribution of managers according to environment and degree of difficulty.

The data in Table 1 and Figure 2 represent the distribution of managers reported in terms of environment and degree of difficulty. The differences observed between groups according to Fisher’s Exact Test were significant (*p* = 0.003), and Bonferroni-corrected Z-tests showed that rural managers reported significantly more often a rather high or moderate level of difficulty than a low one (25%/9% vs. 0.8%) while urban managers reported significantly more often a rather low level of difficulty than moderate or high (99.2% vs. 91%/75%).

The results suggest that the practice environment influences how managers perceive the level of difficulty associated with the context under analysis. Managers in rural areas tend to describe the situation as more demanding, while those in urban areas predominantly perceive it as easier to manage. This difference in perception may reflect distinct structural conditions between the two environments, such as differences in access to resources, digital infrastructure, staff, or support services, which may in turn shape daily managerial experience.

#### 3.3.2. Analysis of the Perception of the Degree of Difficulty in Relation to the Availability

**Figure 3:**
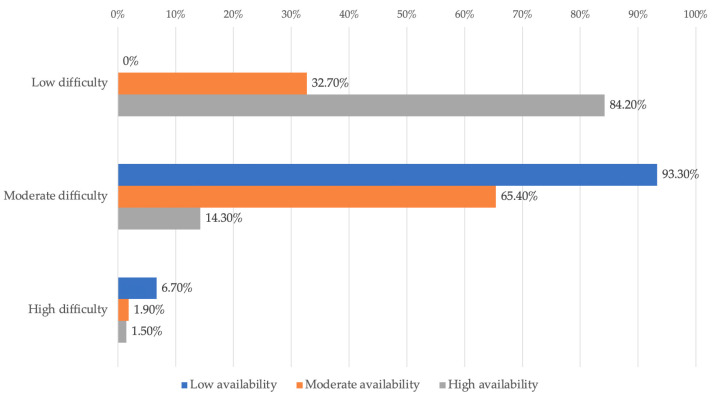
Distribution of managers according to degree of difficulty and degree of availability.

The data in Table 2 and Figure 3 represent the distribution of managers reported in terms of degree of difficulty and degree of availability. The differences observed between groups according to Fisher’s Exact Test were significant (*p* < 0.001), and Bonferroni-corrected Z-tests showed the following: managers who reported low difficulty were significantly more likely to be associated with increased availability (84.2% vs. 32.7%/0%), and managers who reported moderate difficulty were more frequently associated with rather low or moderate availability than high availability (93.3%/65.4% vs. 14.3%).

Managers who perceived the context as easier to manage were more likely to report high availability, while as the difficulty is perceived as moderate, availability shifts to low or at most moderate levels. This association is consistent with change management models, according to which the perception of high effort and increased risk may be associated with lower openness to new initiatives.

#### 3.3.3. Analysis of the Perception of the Degree of Difficulty in Relation to the Use of Digital Instruments

**Figure 4:**
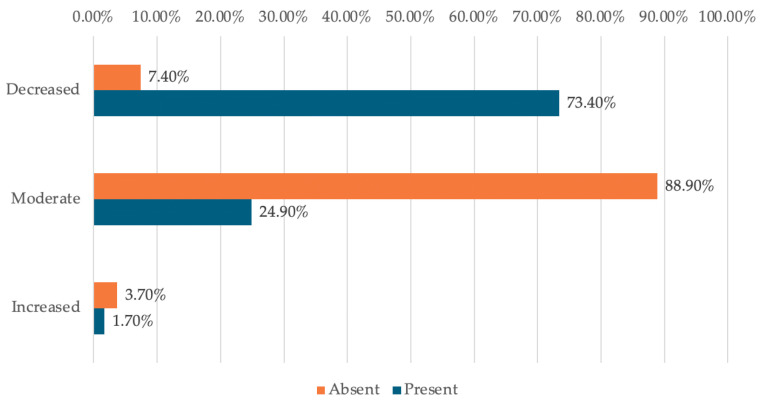
Distribution of managers according to degree of difficulty and use of digital tools.

Data in Table 3 and Figure 4 represent the distribution of managers reported in terms of degree of difficulty and use of digital tools. The differences observed between groups according to Fisher’s Exact Test were significant (*p* < 0.001), and Bonferroni-corrected Z-tests showed the following: managers who reported low difficulty were significantly more likely to use digital tools (73.4% vs. 7.4%), while managers who reported moderate difficulty (88.9% vs. 24.9%) were less frequently associated with the use of digital tools.

This suggests a clear association between managers’ perceived level of difficulty and the reported use of digital tools in their current work. Managers who report a low level of difficulty are clearly those who use digital tools more frequently, while managers who perceive the difficulty as moderate tend to use such solutions less often.

This association can be interpreted in two complementary ways. On the one hand, the use of digital tools may be associated with lower perceived difficulty, possibly through greater workflow standardization, task automation, and improved control over information by standardizing workflows, automating tasks, and increasing control over information. On the other hand, managers who start from a higher perception of difficulty may be more reluctant to adopt new technologies or may work in contexts with insufficient infrastructure, which may contribute to a continuing pattern of non-use.

### 3.4. The Perceived Degree of Availability

#### 3.4.1. Analysis of Availability by Age

**Figure 5:**
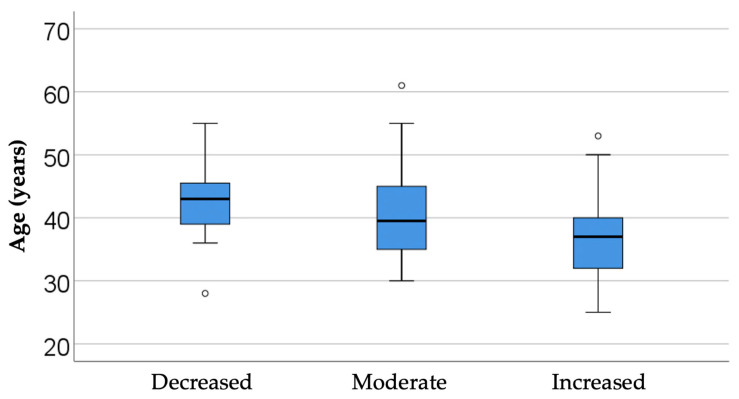
Comparison of managers’ age in relation to their reported availability.

The data in Table 4 and Figure 5 represent the comparison of managers’ age in relation to their reported availability. The differences observed between groups according to the Kruskal-Wallis H test were significant (*p* < 0.001), and Dunn-Bonferroni post-hoc tests show that managers who reported high availability were significantly younger (median = 37, IQR = 32–40) compared to managers who reported moderate availability (median = 39.5, IQR = 35–45) (*p* = 0.005) or low (median = 43, IQR = 38–46) (*p* = 0.002), the differences between the latter two categories were not significant (*p* = 0.494).

From an interpretative point of view, these results are consistent with the literature on innovation diffusion, which describes a greater predisposition of younger groups of professionals to explore and integrate new solutions, while more experienced groups adopt a more cautious attitude, filtering change through the lens of already established working models. However, the differences observed should not be understood as age determinism, but as an average trend: availability is also influenced by other factors, such as organizational culture, previous experiences with digitization, or the level of institutional support.

#### 3.4.2. Analysis of Availability in Relation to Professional Experience

**Figure 6:**
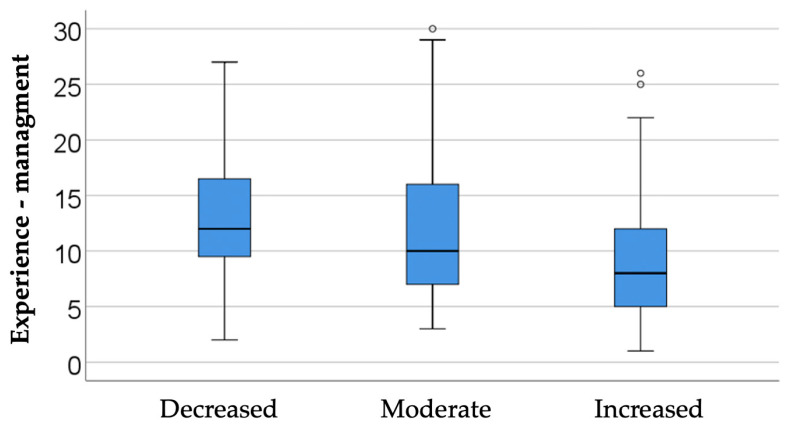
Comparison of managers’ experience in relation to reported availability.

The data in Table 5 and Figure 6 represent the comparison of managers’ experience in relation to reported availability. The differences observed between groups according to the Kruskal-Wallis H test were significant (*p* = 0.001), and Dunn-Bonferroni post-hoc tests show that managers who reported high availability had significantly lower experience (median = 8, IQR = 5–12) compared to managers who reported moderate availability (median = 10, IQR = 7–16) (*p* = 0.018) or low availability (median = 12, IQR = 9–17) (*p* = 0.014), the differences between the latter two categories were not significant (*p* = 0.806).

This distribution suggests that managers in the earlier stages of their careers are generally more open to change, innovation, and the adoption of new tools or organizational models, while managers with long experience develop a more cautious attitude, filtering novelty through the lens of established work routines.

#### 3.4.3. Analysis of Availability in Relation to the Professional Environment

**Figure 7:**
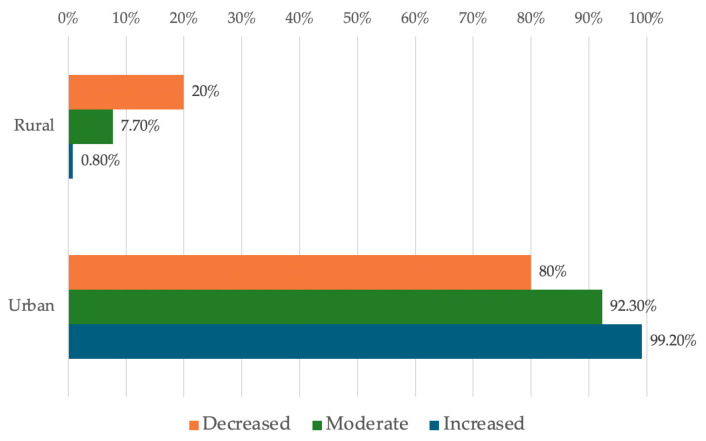
Distribution of managers reported in terms of environment and availability.

The data in Table 6 and Figure 7 represent the distribution of managers reported in terms of environment and availability. The differences observed between groups according to Fisher’s Exact Test were significant (*p* = 0.001), and Bonferroni-corrected Z-tests showed that rural managers reported significantly more often a rather low or moderate level of availability than a high level (20%/7.7% vs. 0.8%) while urban managers reported significantly more often a rather high level of readiness than moderate or low (99.2% vs. 92.3%/80%).

### 3.5. The Use of Digital Tools in Current Practice

#### 3.5.1. Analysis of the Use of Digital Tools by Age Group

**Figure 8:**
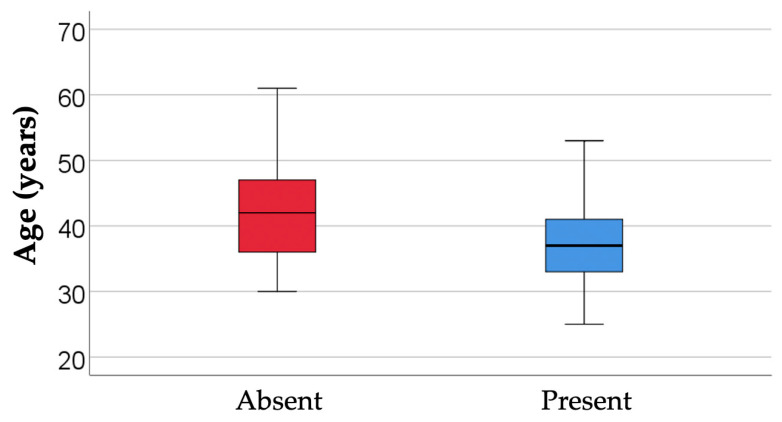
Comparison of managers’ ages in relation to the use of digital tools.

The data in Table 7 and Figure 8 represent the comparison of managers’ ages in relation to the use of digital tools. The differences observed between groups according to the Mann-Whitney U test were significant (*p* = 0.002), so that managers who stated that they currently use digital tools were significantly younger (median = 37, IQR = 33–41) compared to managers who do not use digital tools (median = 42, IQR = 36–47).

This difference is correlated with the hypothesis that increased familiarity with digital media and previous experience with similar technologies favor the adoption of new solutions, while older managers may be more cautious or remain anchored in traditional working patterns. However, the observed relationship should not be interpreted deterministically: age probably interacts with other factors, such as organizational culture, resource availability, access to training, and institutional support.

#### 3.5.2. Analysis of the Use of Digital Tools in Relation to Professional Experience

**Figure 9:**
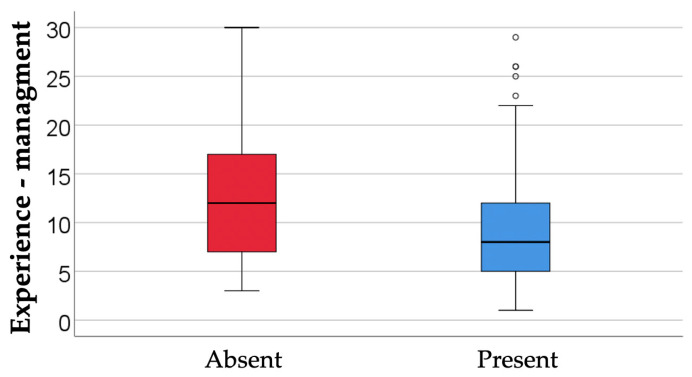
Comparison of managers’ experience with the use of digitalization.

Table 8 and Figure 9 shows a comparison of managers’ experience with the use of digitalization. The differences observed between groups according to the Mann-Whitney U test were significant (*p* = 0.010), with managers who stated that they currently use digital tools having significantly less experience (median = 8, IQR = 5–12) compared to managers who do not use digital tools (median = 12, IQR = 7–17). The results presented in Figure 9 show that the current use of digital tools is more characteristic of managers with less professional experience, while managers with more seniority tend not to use them. This outlines a profile of the “digital manager” at an earlier stage of their career, for whom integrating technology into their daily work is more natural and less disruptive to their usual way of working.

#### 3.5.3. Analysis of the Use of Digital Tools in Relation to Professional Environment

**Figure 10:**
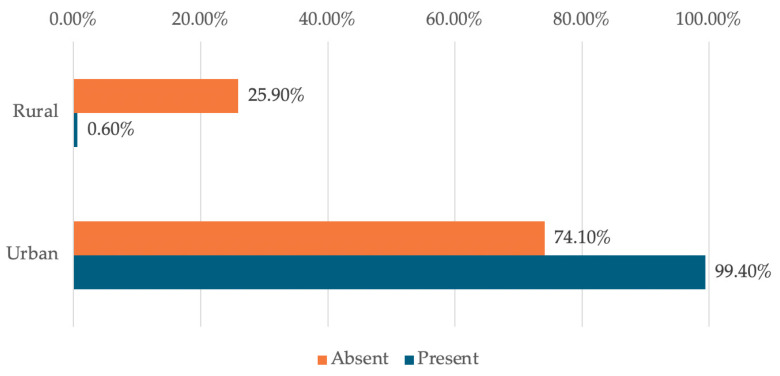
Distribution of managers reported in terms of the environment and the use of digitization.

The data in Table 9 and Figure 10 represent the distribution of managers reported in relation to the environment and the use of digitization. The differences observed between groups according to Fisher’s Exact Test were significant (*p* < 0.001), with urban managers reporting significantly more frequent use of digital tools (99.4% vs. 74.1%).

#### 3.5.4. Description of Interest Scores in Relation to Digitalization

**Figure 11:**
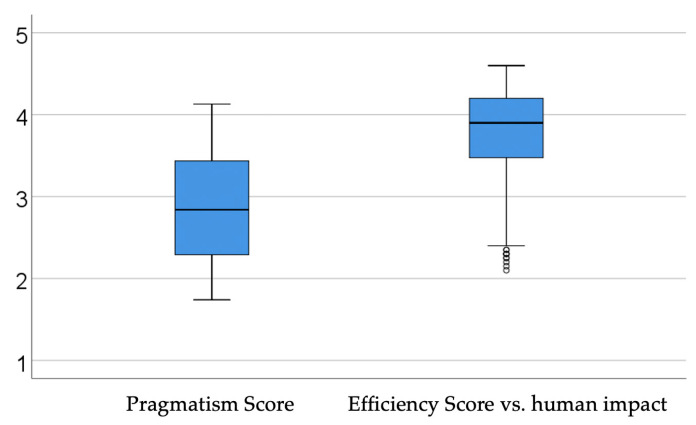
InterestRelation to digitalization.

The data in Table 10 and Figure 11 describe interest scores in relation to digitalization. The observed scores were: Overall managerial pragmatism score regarding digitalization (reflected by the following traits: acceptance of digitalization mainly in terms of efficiency and control, interest in economic and operational impact rather than in the human or innovative side of digitalization; rejection of technological solutions that involve major organizational changes, resistance to change, lack of availability to redesign how the clinic works, caution regarding excessive automation or perceived dependence on technology) (overall, a higher value would indicate a clear pragmatic trait regarding radical digitalization, direct focus on efficiency, and higher concern for clinic sustainability and profitability) (median = 2.84, IQR = 2.29–3.44, showing a moderate level of pragmatism); Overall interest score in the efficiency of digitalization versus its human impact (reflected by the following traits: a specific priority on efficiency and operational optimization, greater concern for efficient management of clinic resources and reduction of costs or wasted time, cautious acceptance of digitalization, greater concern for initial/maintenance costs and security risks, stronger reluctance toward technologies that affect human interaction, avoiding excessive technologization that may reduce direct interaction with patients) (a higher value would indicate a desire for a remote medical process, as simplified as possible) (median = 3.9, IQR = 3.46–4.2, showing a moderate to increased level of interest in efficiency). Construction of the mentioned interest scores is more detailed in the Supplementary Material in Table S3.

The results in Table 10 and Figure 11 outline the profile of a dental practice manager strongly grounded in a logic of digital pragmatism, combined with a clear focus on operational performance. The moderate median value of the pragmatism score suggests that most managers do not reject digitalization itself, but accept it mainly when it clearly serves goals of control, sustainability, and profitability. They seem willing to integrate digital solutions and, by extension, artificial intelligence tools, as long as these do not require deep organizational changes and do not create a level of dependence on technology that is seen as excessive. This attitude may function as a form of risk filter: AI is accepted as long as it supports stability and predictability, not a major change in how the clinic operates. At the same time, the moderate to increased score for efficiency versus human impact indicates that managers clearly prioritize resource optimization, cost reduction, and reducing idle time when they assess the value of digitalization.

## 4. Discussion

### 4.1. Interpretive Analysis of Treatment Subdomains

#### 4.1.1. Analysis of Subdomain—Dispensarization

Within the dental clinical workflow, dispensary care is an essential link in the continuity of care, and from a manager’s perspective, the integration of artificial intelligence may support a transition from a reactive to a more proactive, prevention-focused model. By aggregating clinical, behavioral, and organizational data, models based on digital technologies may support risk stratification and the generation of more personalized recall schedules, adjusting the frequency of check-ups based on the likelihood of recurrence of the treated condition, the complexity of treatments, and the patient’s history. The transmission of medical recommendations and patient education benefits from natural language processing systems that convert treatment plans into easier-to-understand summaries, with visual support and messages tailored to the level of medical knowledge. Digital platforms can create and distribute short videos, quizzes, or personalized reminders and synchronize notifications with patient preferences, which may increase the likelihood of compliance without necessarily inducing information fatigue [37].

#### 4.1.2. Analysis of Subdomain—Treatment Planning

Treatment planning, which involves structuring interventions in stages based on diagnostic information, has attracted increased interest. From the perspective of a dental clinic manager, this may indicate, on the one hand, an awareness of the strategic importance of this stage for clinical outcomes and patient satisfaction and, on the other hand, the classification of this stage as highly important from the perspective of medical staff satisfaction. One plausible explanation, from a management perspective, may be the challenge of introducing advanced technological solutions into an already busy workflow, where staff are under pressure from time constraints and patient expectations. In this context, it becomes essential to take a hybrid approach to the development and presentation of individualized treatment plans, adapting to the needs of the medical and management teams, as well as implementing intuitive and user-friendly digital interfaces that may facilitate the adoption of these solutions without substantially disrupting the organization and efficiency of the clinic [38].

#### 4.1.3. Analysis of Subdomain—Feedback

The analysis of the “Feedback” subdomain indicates, from the perspective of dental clinic managers, the lowest level of interest among the stages evaluated. This reluctance might to be fueled by a pragmatic focus on areas with immediate operational impact (scheduling, dispensary services), the perception of a less clear cost-benefit ratio for the feedback area, and fears regarding patients’ reluctance to complete questionnaires, the representativeness of responses, and the integration of suggestions into the current workflow. However, the potential for improvement is substantial if feedback becomes easier to implement: short tools, distributed across various communication channels, delivered at key moments in the patient’s journey; automatic analysis of comments to extract themes and different types of opinion; dashboards with alert thresholds, designated managers, and remediation deadlines [39].

#### 4.1.4. Analysis of Subdomain—Appointment

From a managerial perspective, scheduling is the cornerstone of operational efficiency: In the appointment domain, AI-supported tools may be used for demand forecasting, appointment-slot optimization, and no-show risk estimation, with the potential to reduce waiting times and improve capacity utilization without increasing pressure on the team. A hybrid approach, combining automation for repetitive tasks with human intervention in sensitive cases, may be more consistent with maintaining trust and the clinician–patient relationship [40].

#### 4.1.5. Analysis of Subdomain—Diagnosis

The moderate to high interest shown in the diagnostic stage may reflect recognition of the potential role of artificial intelligence in supporting rigour and efficiency in the decision-making process, while maintaining the central role of the physician. The integration of digital tools can facilitate the aggregation and structuring of clinical and imaging data, early detection of risk areas, and the proposal of investigation priorities, with favorable effects on standardization and reduction of variability among clinicians. From an operational perspective, preliminary triage and appropriate resource scheduling shorten the time to diagnosis and optimize the allocation of slots for complex cases. In terms of the doctor-patient relationship, presenting results through clear summaries and understandable visual support which may support shared decision-making and improve adherence to the proposed therapeutic conduct. For sustainable implementation, smooth integration into existing workflows, continuous team training, and compliance with confidentiality and transparency rules regarding data use are essential [41].

### 4.2. Practical Aspects of the Results

Beyond their descriptive value, the findings of the present exploratory study may also be considered from a practical perspective, particularly in relation to how managers perceive difficulty, express availability for change, use digital tools in current practice, and position themselves toward AI-related digitalization. Taken together, these dimensions may offer a useful preliminary picture of how organizational readiness could take shape in dental settings, while also suggesting that implementation processes are likely to remain context-dependent rather than uniform across environments or managerial profiles.

#### 4.2.1. The Perceived Degree of Difficulty

A first practical layer of interpretation concerns the perceived degree of difficulty, as this dimension may reflect how manageable organizational change appears within different professional contexts. In an exploratory framework such as the present one, this perception may be especially relevant because it can influence not only attitudes toward digitalization, but also the degree to which managers feel able to engage with change in everyday practice.

Analysis of the Perception of the Degree of Difficulty in Relation to the Professional Environment—In terms of practical implications, these findings support the idea that strategies for implementing organizational change, including those related to digitization and AI, cannot be standardized. Based on the observed pattern, managers in rural areas may benefit from additional support measures (training, mentoring, technical and logistical support), while in urban areas, the focus may be more on refining and optimizing already functional flows. Overall, the analysis highlights the importance of a differentiated, context-sensitive approach to designing managerial interventions in dentistry.

When viewed alongside availability, perceived difficulty may become even more informative, as it may help clarify whether organizational barriers are likely to weaken openness toward change or whether they might be partially reduced through targeted implementation support. In this sense, the relationship between these two variables may offer a more nuanced understanding of managerial readiness.

Analysis of the Perception of the Degree of Difficulty in Relation to the Availability—From a practical perspective, the results indicate that in order to increase managers’ availability, it is not enough to simply promote the benefits; it may also be important to address perceived difficulties by clarifying processes, providing technical support, and offering adequate training by clarifying processes, providing technical support, and offering adequate training.

This perspective may also be considered in relation to the current use of digital instruments, since familiarity with digital tools could plausibly influence the way change is interpreted and operationalized. Although the present study does not allow causal inferences, the observed association may still be relevant from a practical planning perspective.

Analysis of the Perception of the Degree of Difficulty in Relation to the Use of Digital Instruments—The results suggest that greater use of digital tools may be linked to lower perceived difficulty; however, this interpretation remains associative and should not be read as evidence of a directional effect.

#### 4.2.2. The Perceived Degree of Availability

If perceived difficulty reflects one side of organizational adaptation, the perceived degree of availability may offer a complementary perspective by indicating how willing managers may be to engage with digital transformation. In this respect, availability may be seen as a more proactive dimension of readiness, while still remaining influenced by multiple personal and contextual factors.

One such factor may be age, which, in an exploratory sense, can be viewed as a potential marker of career stage, prior exposure to digital change, and differing attitudes toward organizational innovation. Examining availability across age groups may therefore contribute to a more nuanced understanding of managerial openness.

Analysis of Availability by Age—Overall, the data suggests that training and motivation strategies should also be calibrated according to the age and career stage of managers in order to capitalize on both the openness of younger generations and the experience of those with seniority.

Closely related to age, professional experience may add another interpretive layer, as it may shape both confidence in managerial decision-making and attachment to pre-existing organizational routines. In this context, the observed patterns may be useful not as deterministic profiles, but as preliminary indications of how different stages of professional development could influence openness to change.

Analysis of Availability in Relation to Professional Experience—From an interpretative perspective, the results are consistent with theories of innovation diffusion, which place professionals who are less anchored in old models of practice among the “early adopters.” However, the relationship should not be viewed as a determinism of seniority: availability is also influenced by institutional factors, organizational culture, and previous experiences with digitization. Consequently, implementation strategies should capitalize on the enthusiasm of younger managers and, at the same time, actively involve experienced managers in order to integrate their knowledge capital and reduce resistance to change.

At the same time, availability may also be shaped by the broader professional environment, where access to infrastructure, support services, and organizational resources may influence how feasible change appears in day-to-day practice. In this sense, the environment may not determine managerial availability, but it may provide more or less favorable conditions for its expression.

Analysis of Availability in Relation to the Professional Environment—This distribution suggests that rural areas are perceived as a less favorable context for embracing change, either due to organizational limitations (limited resources, insufficient digital infrastructure, reduced access to technical support) or through the accumulation of previous experiences that have reinforced a higher level of caution. In urban areas, where access to technology, maintenance services, and professional networks is generally easier, managers’ stated availability is significantly higher, which may accelerate the adoption and consolidation of digital workflows.

#### 4.2.3. The Use of Digital Tools in Current Practice

If availability may be regarded as an expression of declared readiness, the actual use of digital tools may offer a more concrete indication of how far digitalization has already entered routine managerial practice. This operational dimension may be especially relevant, because it moves the discussion from attitudes and perceptions toward observable patterns of current use.

A first point of entry here may be age group, as different career generations may not only differ in their openness to digitalization, but also in the extent to which they have incorporated digital instruments into everyday work. In an exploratory study, such differences may help contextualize the practical conditions of implementation.

Analysis of the Use of Digital Tools by Age Group—From a practical perspective, the results suggest that training programs and implementation strategies should take into account the career stage of managers, specifically supporting those with more experience to facilitate the adoption and effective use of digital tools.

Professional experience may again provide additional nuance, particularly because longer experience can be associated both with valuable managerial capital and with more stable organizational habits. The practical relevance of this relationship may lie in the possibility of designing implementation models that do not simply replace older routines, but create bridges between different forms of expertise.

Analysis of the Use of Digital Tools in Relation to Professional Experience—Conversely, long experience seems to be associated with maintaining traditional routines of organization and control, which can lead to greater inertia towards technological change, even if it promises increased efficiency. This pattern suggests a generational gap in the adoption of digitalization at the managerial level and indicates that implementation strategies could benefit from “reverse mentoring” approaches: younger managers, familiar with digital tools, can become resources for their experienced colleagues, thus facilitating a smoother transition to digitized workflows.

Beyond age and experience, the professional environment may also shape the real-world conditions under which digital tools are adopted and sustained. This point is particularly relevant in the context of organizational digitalization, because uneven implementation conditions may reinforce broader disparities in managerial capacity and workflow modernization.

Analysis of the Use of Digital Tools in Relation to Professional Environment—In terms of practical implications, the results show a potential digital divide between urban and rural areas, which may have implications for the reported ability to implement digitized workflows and, potentially, artificial intelligence-based tools. In this context, public policy strategies and professional interventions should prioritize support for rural units—through investments in infrastructure, training programs, and technical support mechanisms—in order to reduce access differences and avoid the consolidation of deeply unequal models of practice from a digitization perspective.

#### 4.2.4. Description of Interest Scores in Relation to Digitalization

Taken together, the previous findings may outline the practical background against which managers evaluate digitalization more broadly, including AI-related tools. However, declared interest in digitalization may add a further strategic dimension, as it may reflect not only current use, but also the conditions under which managers might consider future adoption acceptable, useful, or sustainable.

In this final layer of interpretation, the focus shifts from existing digital habits toward the way managers appear to frame AI as a potential organizational instrument. In an exploratory sense, these scores may be especially useful because they suggest not only whether AI is viewed favorably, but also under what conditions it might be integrated into practice without disrupting existing professional and relational structures.

Description of Interest Scores in Relation to Digitalization—Their interest in AI seems mainly practical: algorithms are valued for their ability to improve scheduling, standardize workflows, and deliver clear performance indicators. Still, this focus on efficiency is paired with clear caution toward solutions that could negatively affect direct interaction with the patient, or that involve initial and maintenance costs that are hard to control. The result is a careful balance: AI is desired for what it adds in operational terms, but it is viewed with concern when it may “dilute” the relational side of medical care. From a managerial perspective, this profile suggests that adoption of AI in workflows will be mainly gradual and conditional, not disruptive. Managers will be open to AI solutions that fit existing processes (e.g., modules integrated into the management software already used, data analysis tools that do not require a major reorganization of activity), rather than platforms that require a deep redesign of roles and responsibilities. At the same time, the focus on efficiency can support implementation if AI projects are presented clearly in terms of time savings, fewer errors, and workflow optimization, together with clear guarantees on data security and protection of the clinician–patient relationship. Overall, the scores describe a type of pragmatism focused on efficiency, but also sensitive to risks and to the human side of care, which does not block AI adoption, but instead shapes its pace and form in dental practices. The practical implication is that strategies for introducing AI must be built not only as technology projects, but also as change management projects: with clear operational targets that can be measured, pilot steps, feedback mechanisms, and clear guarantees that digitalization remains a tool to support, not replace, clinical judgment and the clinician–patient relationship.

Overall, these practical aspects of the results may suggest that digital transformation in dentistry is unlikely to function as a purely technical process. Rather, within the limits of this exploratory study, it appears more likely to unfold as a gradual organizational transition shaped by perceived difficulty, declared availability, existing digital habits, and the way managers weigh efficiency against relational, ethical, and structural concerns. From this perspective, implementation may be more sustainable when it is approached not only as the introduction of new tools, but also as a context-sensitive process of organizational adaptation.

### 4.3. AI in Dentistry and Perspectives for Workflow Optimization

As dentistry and dental practice management enter a more mature phase of digital change, recent literature shows that artificial intelligence is no longer only a tool for limited clinical support, but is starting to reshape the structure of the whole workflow and governance at clinic level. A study carried out among dentists in Romania shows a high interest in integrating AI into daily practice, but also that the decision to implement it is strongly filtered through organizational feasibility, costs, and how clear the legal framework is, which places the clinic manager at the center of the adoption process [31]. In parallel, research conducted in Germany shows that the level of digitalization and openness to advanced technologies, including AI-based systems for documentation, scheduling, image analysis, or patient communication are strongly influenced by the organization’s “technology readiness”, clinic size, and the existence of an explicit innovation strategy. This suggests that AI may increase differences between practices if targeted support policies for small and medium units are not developed [42]. Narrative and systematic reviews on AI applications in dentistry describe not only benefits in diagnosis and planning, but also a significant potential for automating administrative tasks (documentation, managing electronic records, scheduling optimization, inventory management), with a direct impact on organizational efficiency and on shifting clinician time toward high-value activities. Other studies show that, although clinicians recognize the potential of AI and modern technologies, the real use of advanced digital tools in practice management and workflow organization remains limited. The main barriers are knowledge gaps, lack of structured training, and fear of digital stress, which underlines the need for training programs dedicated to managers and leadership teams [43,44]. From an educational and organizational culture perspective, the growing preference of students for fully digital workflows in prosthodontics, and the complexity of post-endodontic prosthetic decisions described in recent studies, suggest that future generations of clinicians will be better prepared to work in deeply digital environments. In such settings, AI can be linked with CAD/CAM, 3D imaging, and decision-support systems to standardize and make clinical pathways more transparent [45,46]. This direction is supported by book-chapter type contributions, such as a recent IntechOpen chapter on artificial intelligence in dental education, which highlights the role of AI-managed learning platforms in reshaping curricula and teaching [47]. The authors describe the move from simple expert systems to adaptive platforms that personalize learning by analyzing student data, offering real-time feedback, and dynamically adjusting content, using tools such as natural language processing, automated assessment, and intelligent tutors. They also discuss challenges related to data protection, algorithm bias, interoperability, and ethical issues, as well as the effects on the role of teaching staff, continuing education, and institutional policies. This type of analysis provides a useful framework for developing adaptive and ethically responsible learning environments, directly relevant to how dental schools and clinics prepare and support teams as AI-based workflows expand.

Beyond imaging and scheduling applications, clinically relevant AI use cases are also emerging in medication-safety workflows. In oral surgery, large language models have recently been compared with oral surgeons in detecting clinically relevant drug–drug interactions, illustrating both the potential utility of AI-assisted screening and the need for careful human oversight in real decision-support scenarios. More broadly, recent work on AI-powered drug–drug interaction research highlights the importance of validation, vulnerable populations, and regulatory governance for the safe and responsible implementation of such systems in clinical practice [48,49].

To achieve more effective human resource management and to allocate appropriate roles within a team for specific tasks, it is essential to understand the competencies and capabilities of each team member. Accurate competency mapping requires a systematic and precise identification of these attributes. One widely used approach for profiling individual behavioral tendencies and interpersonal dynamics inside teams is the DISC framework, which can support role alignment, task distribution, and overall team performance in clinical settings.

### 4.4. DISC Model: Driven Optimization of Clinical and Digital Workflows in Dentistry

Although DISC-related constructs were not directly measured in this study, the model may offer a useful conceptual lens through which the observed patterns of attitudes toward AI adoption can be interpreted. In this sense, its role in the present manuscript is not to provide an empirical conclusion, but rather to serve as a hypothesis-generating framework that may inform future research and implementation planning.

The DISC model has its roots in the work of psychologist William Moulton Marston, who, in “Emotions of Normal People” (1928), proposed a scheme of human behavior based on four emotional–behavioral patterns: Dominance, Inducement/Influence, Submission/Steadiness, and Compliance/Conscientiousness, each linked to specific patterns of communication, decision-making, and relation to rules and tasks. It is worth noting that, unlike later psychometric models, Marston’s original theory was not designed as a clinical diagnostic tool, but as a map of how individuals react to their environment, perceived as favorable or unfavorable, and to needs for control or adaptation [50]. In organizations, including those in healthcare, the DISC model is used mainly in three directions:

***Personal development and leadership:*** In leadership development programs, the DISC model is used to support self-awareness of leaders’ behavioral style and to show how it influences decisions, communication, and team management. Understanding one’s own profile (for example, action focus typical for D, relationship focus typical for I, stability focus typical for S, and rigor focus typical for C) allows a leader to consciously adapt behavior to the needs of colleagues. In this way, DISC becomes a reflection tool that supports a shift from rigid leadership to a situational and flexible style, especially in periods of organizational change such as digitalization.

***Teamwork and conflict management:*** At team level, DISC profiles offer a shared language to describe differences between members in neutral terms, work pace, need for clarity, risk appetite, and focus on relationships versus tasks. Instead of viewing tensions as “personality problems”, they can be understood as style differences, which reduces personal blame and opens space for negotiation and complementarity. In this sense, DISC helps build collaboration, where roles, expectations, and preferred communication styles are discussed explicitly rather than left as assumptions.

***General fit between role and style:*** Regarding the person—role link, DISC is not used as a “hard” selection test, but as an orienting guide for discussing compatibility between behavioural style and job demands. For example, roles involving quick decisions, negotiation, and risk-taking may benefit from a stronger D component, while roles requiring systematic analysis, compliance, and attention to detail may fit better with a stronger C profile. Importantly, this fit is seen as dynamic and developable: individuals can adjust behavior over time, and managers can redesign tasks to use team strengths without turning DISC into a rigid inclusion/exclusion rule.

In the medical field, studies on managerial care settings and clinical teams show that using a DiSC-type tool can improve communication and collaboration. Keogh et al. used DiSC Classic in communication-skills workshops for 3396 nurse managers, showing that most participants had dominant profiles in Dominance and Conscientiousness, and that understanding their own style helped them explain their reactions in work interactions, adjust behavior consciously, and build stronger relationships with their teams. The authors emphasize that tools like DiSC, which are relatively easy to administer and have satisfactory psychometric features, can be a useful complement in leader selection and development, adding information beyond the classic interview. In dentistry, Medina proposes using DISC profiling to personalize the patient journey and strengthen the team, arguing that in an intense clinical context focused on technical performance, a organized framework for understanding behavioral styles can support effective communication with patients, improve treatment plan acceptance, increase patient satisfaction, and support team harmony, strengthening the relevance of the DISC model in dental practice as well [51].

**Figure 12:**
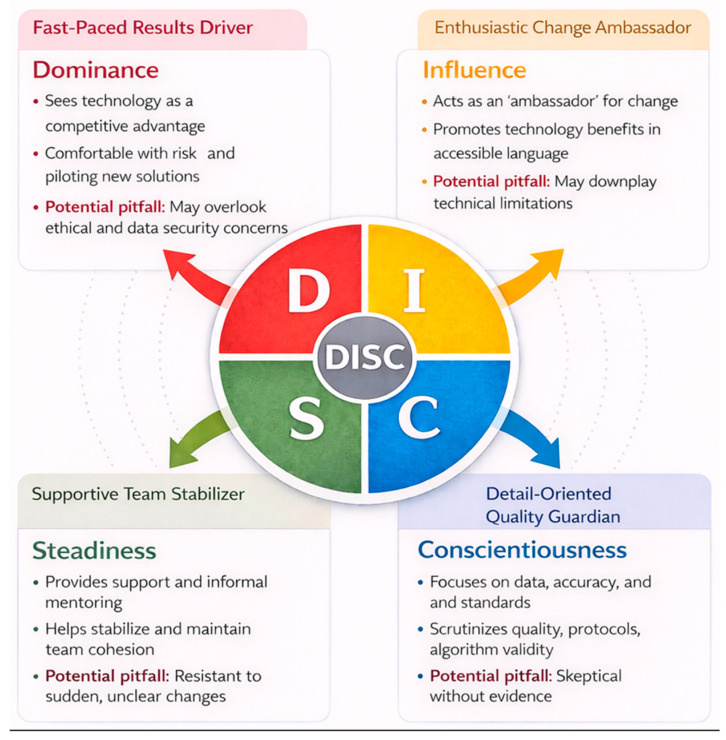
Model adapted to AI integration in dental practice.

In the context of integrating artificial intelligence into dental practice, the four dimensions of the DISC model may be tentatively used as a conceptual lens for interpreting distinct patterns of how individuals might relate to technology, risk, and organizational change (Figure 12). The literature describes the D (Dominance) profile as focused on results, fast decisions, risk-taking, and a need for control. In the shift toward AI-augmented workflows, this profile tends to see technology as a competitive advantage and a performance accelerator for the clinic. People with high D scores tolerate uncertainty better, are willing to pilot new solutions, and more easily accept implementation decisions even when not all data are available. However, this strong action focus can, without counterbalances, lead to underestimating ethical issues, data security concerns, or the impact on the clinician–patient relationship, which may be seen as “brakes” on innovation. For this reason, D profiles are valuable in leading AI projects, but should be balanced by other styles, especially C and S, in decision-making structures. The I (Influence) profile is characterized by sociability, enthusiasm, relationship focus, and strong persuasion skills. In AI adoption, individuals with a dominant I style can act as “ambassadors” of change: they explain technology benefits in accessible language to both colleagues and patients, build positive narratives (“AI frees time for patient interaction”, “it reduces routine paperwork and errors”), and help create a climate of curiosity toward new digital tools. They are often natural fits for internal training, system presentation workshops, and multi-channel communication. On the other hand, an imbalance in favor of this style—without the careful analysis typical of C profiles—can lead to a more “image-based” than “substance-based” promotion of AI, with risks of overpromising and downplaying technical limits or algorithm uncertainty. People with an S (Steadiness) profile seek stability, consistency, and harmony at work, and are strongly oriented toward support, collaboration, and team cohesion. In AI integration, these traits translate into an important contribution to stabilizing processes after the initial implementation phase. S individuals often support more hesitant colleagues, provide informal mentoring, and create a supportive climate, essential for a major technological change to be adopted by the whole team. Once they understand and accept the reasons for new procedures, they apply them consistently and can become guardians of practice consistency. However, if change is seen as abrupt, threatening, or poorly explained, S styles may show resistance, expressed as a wish to keep the “old way” of working. Therefore, early involvement in co-designing new workflows and explicit recognition of their stabilizing role are decisive for success. The C (Conscientiousness) profile is defined by cognitive rigor, attention to detail, focus on rules, procedures, and standards, and a strong need for clarity and evidence before adopting change. In a digitalized clinic, these features make C-style individuals good candidates for roles in data governance and quality control: monitoring completeness and accuracy of data entered into AI systems, documenting and updating usage protocols, and critically reviewing algorithm performance (error rates, edge cases, situations where AI recommendations differ from clinician judgment). At the same time, a strong C component can lead to skepticism or reluctance if there is not enough information about algorithm validation, training datasets, or decision traceability. As a result, involving these people in selection, evaluation, and audit phases of AI solutions and giving them access to technical documentation and performance data is not only recommended, but essential to turn justified skepticism into constructive vigilance and strong patient and organizational safeguards.

The results of this study indicate that, from an administrative perspective, the digital transformation of dental clinics is driven mainly by a strong orientation toward efficiency, productivity, and resource optimization. Managers do not view digital workflows and artificial intelligence as simple “technology add-ons”, but as real strategic levers for reorganizing processes, reducing redundancies, and increasing working capacity, while keeping an adequate level of clinical quality. This view reflects less a superficial enthusiasm for innovation and more a coherent managerial way of thinking, in which digitalization and AI are integrated into competitiveness, economic sustainability, and performance control. However, such a strong focus on efficiency also highlights, in return, the need to balance operational gains with the human, relational, and ethical aspects of care. If AI is shaped only through time, cost, and volume indicators, there is a risk that digital transformation becomes a technical process focused on software and equipment, and less a socio-technical project centered on the team and the patient. Our results suggest that meaningful AI implementation in dental clinics may require a redefinition of the managerial role: from “resource administrator” to leader of digital change, able to design workflows together with dentists, assistants, and administrative staff, and to use tools such as the DISC model for a smart and balanced distribution of responsibilities within the team. In this logic, a dual approach to digitalization policies and strategies in dentistry becomes essential. On one hand, a strong European framework is needed to set standards for interoperability, data security, and responsible use of artificial intelligence in healthcare. On the other hand, national implementation mechanisms are indispensable, adapted to the real conditions of practices and clinics, especially small and medium ones. For managers, this means access to operational guides, risk assessment and management tools, contract templates, and examples of good practice that turn broad digital strategies into concrete operational decisions in each organization. At the same time, the results of this study underline the need to align managerial and clinical training with the digital era. Dental clinic managers, regardless of age, experience, and work setting, need clear skills in digital leadership, basic AI literacy, data governance, and change management. These skills cannot remain implicit or be left only to daily experience, but must be built into university and postgraduate programs, and into continuing medical education modules that include both technical components (understanding limits, risks, and AI potential) and human components (communication, team management, using DISC-type frameworks to harmonize work styles). Only under these conditions can the managerial focus on efficiency be turned into real time and energy gains for the clinician–patient relationship, rather than simply increasing work pace and pressure on staff.

Accordingly, the DISC model should not be interpreted here as an empirical conclusion derived from the study data, but rather as a potential framework for future research and implementation planning in the context of AI integration in dental practice.

### 4.5. Limitations

This study also has limitations. The number of participants is relatively small, their practice setting is limited, and the research was carried out only in Bucharest and nearby areas, which limits how far conclusions can be generalized nationally or internationally. In addition, the field analyzed, digitalization and AI integration in dental practice, changes very quickly, so the picture captured by this study may become partly outdated within a relatively short time. A key limitation of this study is the marked imbalance in respondents’ area of residence, with 96% of managers from urban settings, which likely reduced the interpretability and added value of urban–rural comparisons and limits the generalizability of findings to rural practice contexts. Another limitation is that about half of the respondents had 4–10 years of experience, which made the experience groups uneven and limited the clarity of comparisons between managers with different levels of experience. A further limitation is the age imbalance in the sample, with 34% of respondents aged 36–40 years; this clustering may reflect that mid-career professionals are both more likely to hold managerial roles and more reachable through the professional/digital recruitment channels used in this study, thereby reducing the representativeness of other age brackets and limiting the robustness of age-based comparisons. In this context, future work should build on these results and extend research on AI and its integration in the daily work of managers in healthcare through more related studies that explore different clinic types, organizational models, and socio-economic contexts. To better understand the world in which medical and administrative staff work, it is not enough to study the present; we must also address future challenges in a systematic way, including scenarios about technology evolution, regulations, and patient expectations. Despite these limits, the results send a clear message: the digital transformation of dental clinics is not only a technology issue, but one of managerial vision, organizational structure, and a culture of learning. If this strong efficiency orientation identified among managers is guided by solid ethical frameworks, well-designed policies, and systematic investment in skills development, artificial intelligence and digital workflows will not reduce dentistry to a volume industry, but will support a practice that is more precise, more predictive, and, in the end, more human—fit for the demands of the digital era.

## 5. Conclusions

From an administrative perspective, the integration of artificial intelligence into dental clinics is no longer a distant promise, but a present-day turning point that is changing how workflows are structured, how resources are allocated, and how decision-making responsibility is defined. However, the impact of AI is not primarily technological, but deeply organizational. Successful implementation is likely to be associated with the clinic’s organizational culture, especially its ability to support psychological safety, continuous improvement, and data-driven governance, rather than treating digital tools as isolated add-ons.

In this context, sustainable adoption requires an appropriate level of team maturity, reflected in the team’s capacity to adapt, communicate effectively, and maintain clinical consistency under changing operational demands. This transition may be further supported through a reverse mentoring strategy, in which digitally skilled team members support senior clinicians and managers through navigating new platforms, automation, and AI-enabled workflows, thereby improving cohesion and reducing resistance to change. Taken together, these findings should be interpreted as preliminary and hypothesis-generating, offering an initial basis for future confirmatory research on AI implementation in dental practice.

In this context, the risk framework identified in the present study should be understood as a structured managerial approach in which AI adoption is evaluated not only through its expected efficiency gains, but also through its potential impact on patient safety, data protection, regulatory compliance, workflow disruption, and the clinician-patient relationship. From this perspective, successful implementation does not rely on rapid or indiscriminate digitalization, but on phased integration, human oversight, staff training, and continuous monitoring to ensure that operational benefits do not come at the expense of clinical quality, trust, or organizational stability.

Ultimately, AI and digitalization can turn efficiency pressures into a meaningful opportunity: freeing time for the clinician–patient relationship, improving predictability in scheduling and resource planning, and reinforcing compliant data stewardship. However, this potential is more likely to be realized through structured change management, where uncertainty is approached not as disruption, but as a catalyst for learning, alignment, and innovation. As Simon Sinek states, “*The opportunity for creativity begins the moment we don’t know what we’re doing*.”

## Figures and Tables

**Table 1:** Distribution of the managers according to professional environment and degree of difficulty.

*Environment/Difficulty (Nr., %)*	*Decreased*	*Moderate*	*Increased*	*p* *
Rural	1 (0.8%)	6 (9%)	1 (25%)	0.003
Urban	128 (99.2%)	61 (91%)	3 (75%)	

* Fisher’s Exact Test, Fisher-Freeman-Halton statistic = 11.642, Cramer’s V = 0.249.

**Table 2:** Distribution of the managers according to the degree of availability and degree of difficulty.

*Availability/* *Difficulty (Nr., %)*	*Low Availability*	*Moderate Availability*	*High Availability*	*p* *
Low difficulty	0 (0%)	17 (32.7%)	112 (84.2%)	<0.001
Moderate difficulty	14 (93.3%)	34 (65.4%)	19 (14.3%)	
High difficulty	1 (6.7%)	1 (1.9%)	2 (1.5%)	

* Fisher’s Exact Test, Fisher-Freeman-Halton statistic = 77.177, Cramer’s V = 0.430.

**Table 3:** Distribution of the managers according to the degree of difficulty and usage of digital instruments.

*Usage/Difficulty (Nr., %)*	*Absent*	*Present*	*p* *
Decreased difficulty	2 (7.4%)	127 (73.4%)	<0.001
Moderate difficulty	24 (88.9%)	43 (24.9%)	
Increased difficulty	1 (3.7%)	3 (1.7%)	

* Fisher’s Exact Test, Fisher-Freeman-Halton statistic = 45.136, Cramer’s V = 0.473.

**Table 4:** Comparison of managers’ age according to availability.

*Availability*	*Mean ± SD*	*Median (IQR)*	*Mean Rank*	*p* *
Decreased (N = 15)	42.6 ± 6.26	43 (38–46)	142.03	<0.001
Moderate (N = 52)	40.35 ± 6.56	39.5 (35–45)	118.50	
Increased (N = 133)	36.82 ± 5.82	37 (32–40)	88.78	

* Kruskal-Wallis H Test, H(2) = 18.265, η^2^ = 0.082.

**Table 5:** Comparison of managers’ experience according to availability.

*Availability*	*Mean ± SD*	*Median (IQR)*	*Mean Rank*	*p* *
Decreased (N = 15)	13.8 ± 7.14	12 (9–17)	135.07	0.001
Moderate (N = 52)	11.81 ± 6.8	10 (7–16)	118.35	
Increased (N = 133)	8.79 ± 5.15	8 (5–12)	90.41	

* Kruskal-Wallis H Test, H(2) = 13.348, η^2^ = 0.057.

**Table 6:** Distribution of the managers according to the degree of availability and environment.

*Availability/* *Environment (Nr., %)*	*Decreased*	*Moderate*	*Increased*	*p* *
Rural	3 (20%)	4 (7.7%)	1 (0.8%)	0.001
Urban	12 (80%)	48 (92.3%)	132 (99.2%)	

* Fisher’s Exact Test, Fisher-Freeman-Halton statistic = 12.834, Cramer’s V = 0.278.

**Table 7:** Comparison of managers’ age according to usage of digital tools.

*Use of Digital Tools*	*Mean ± SD*	*Median (IQR)*	*Mean Rank*	*p* *
Absent (N = 27)	42.44 ± 7.62	42 (36–47)	132.41	0.002
Present (N = 173)	37.5 ± 5.86	37 (33–41)	95.52	

* Mann-Whitney U Test, U = 1474, z-score = −3.085, r = 0.218.

**Table 8:** Comparison of managers’ experience according to usage of digital tools.

*Use of Digital Tools*	*Mean ± SD*	*Median (IQR)*	*Mean Rank*	*p* *
Absent (N = 27)	13.59 ± 7.94	12 (7–17)	127.17	0.010
Present (N = 173)	9.38 ± 5.45	8 (5–12)	96.34	

* Mann-Whitney U Test, U = 1615, z-score = −2.579, r = 0.182.

**Table 9:** Distribution of the managers according to the environment and usage of digital instruments.

*Usage/Environment (Nr., %)*	*Absent*	*Present*	*p* *
Rural	7 (25.9%)	1 (0.6%)	<0.001
Urban	20 (74.1%)	172 (99.4%)	

* Fisher’s Exact Test, **χ^2^(1)** = 39.078, Cramer’s V = 0.442.

**Table 10:** Description of Interest Scores in Relation to Digitalization.

*Score*	*Mean ± SD*	*Median (IQR)*
Score–Pragmatism	2.91 ± 0.66	2.84 (2.29–3.44)
Score–Efficiency vs. human impact	3.75 ± 0.6	3.9 (3.46–4.2)

## Data Availability

The original contributions presented in this study are included in the article and supplementary material. Further inquiries can be directed to the corresponding author.

## Supplementary Materials

The following supporting information can be downloaded at: https://www.mdpi.com/article/10.3390/dj14040206/s1, Table S1: Distribution of the managers according to the answers in the survey; Table S2: Construction of subdomains scores analyzed in the study; Table S3. Construction of interest scores regarding digitalization.
