# Supplementary material for: Administrative Perspectives on Digital Workflow Transformation and Artificial Intelligence Implementation in Dental Clinics
Source: Dent J (Basel). 2026 Apr 2;14(4):206. doi: 10.3390/dj14040206 (PMC13114997; doi:10.3390/dj14040206)
Supplement: Supplementary file 1 [file dentistry-14-00206-s001.zip › dentistry-4146457-supplementary.pdf]

Table S1. Distribution of the managers according to the answers in the survey

Item/Interest (Nr., %)	Minimum	Low	Moderate	High	Maximum
**8A**	25 (12.5%)	46 (23%)	56 (28%)	**65 (32.5%)**	8 (4%)
**8B**	6 (3%)	10 (5%)	17 (8.5%)	60 (30%)	**107 (53.5%)**
**8C**	64 (32%)	**70 (35%)**	34 (17%)	15 (7.5%)	17 (8.5%)
**8D**	45 (22.5%)	**46 (23%)**	40 (20%)	35 (17.5%)	34 (17%)
**8E**	**60 (30%)**	28 (14%)	53 (26.5%)	25 (12.5%)	34 (17%)
**9A**	26 (13%)	45 (22.5%)	**56 (28%)**	36 (18%)	37 (18.5%)
**9B**	6 (3%)	20 (10%)	30 (15%)	45 (22.5%)	**99 (49.5%)**
**9C**	43 (21.5%)	**56 (28%)**	34 (17%)	44 (22%)	23 (11.5%)
**9D**	**67 (33.5%)**	61 (30.5%)	28 (14%)	30 (15%)	14 (7%)
**9E**	**58 (29%)**	18 (9%)	52 (26%)	45 (22.5%)	27 (13.5%)
**10A**	27 (13.5%)	**70 (35%)**	64 (32%)	24 (12%)	15 (7.5%)
**10B**	2 (1%)	7 (3.5%)	15 (7.5%)	38 (19%)	**138 (69%)**
**10C**	**71 (35.7%)**	46 (23.1%)	47 (23.6%)	25 (12.6%)	10 (5%)
**10D**	54 (27.1%)	38 (19.1%)	28 (14.1%)	**63 (31.7%)**	16 (8%)
**10E**	46 (23.4%)	38 (19.3%)	45 (22.8%)	**48 (24.4%)**	20 (10.2%)
**11A**	33 (16.5%)	49 (24.5%)	**60 (30%)**	36 (18%)	22 (11%)
**11B**	5 (2.5%)	13 (6.5%)	16 (8%)	46 (23%)	**120 (60%)**
**11C**	**105 (52.5%)**	51 (25.5%)	17 (8.5%)	17 (8.5%)	10 (5%)
**11D**	14 (7%)	38 (19%)	50 (25%)	**67 (33.5%)**	31 (15.5%)
**11E**	43 (21.5%)	49 (24.5%)	**57 (28.5%)**	34 (17%)	17 (8.5%)
**12A**	7 (3.5%)	35 (17.5%)	56 (28%)	**73 (36.5%)**	29 (14.5%)
**12B**	22 (11%)	34 (17%)	**69 (34.5%)**	55 (27.5%)	20 (10%)
**12C**	67 (33.5%)	23 (11.5%)	10 (5%)	14 (7%)	**86 (43%)**
**12D**	34 (17%)	**46 (23%)**	44 (22%)	44 (22%)	32 (16%)
**12E**	**70 (35%)**	62 (31%)	21 (10.5%)	14 (7%)	33 (16.5%)
**13A**	69 (34.5%)	**74 (37%)**	32 (16%)	18 (9%)	7 (3.5%)
**13B**	11 (5.5%)	41 (20.5%)	**81 (40.5%)**	44 (22%)	23 (11.5%)
**13C**	2 (1%)	4 (2%)	14 (7%)	31 (15.5%)	**149 (74.5%)**
**13D**	40 (20%)	29 (14.5%)	27 (13.5%)	**91 (45.5%)**	13 (6.5%)
**13E**	**78 (39%)**	52 (26%)	46 (23%)	16 (8%)	8 (4%)
**14A**	4 (2%)	31 (15.5%)	**65 (32.5%)**	39 (19.5%)	61 (30.5%)
**14B**	16 (8%)	15 (7.5%)	29 (14.5%)	49 (24.5%)	**91 (45.5%)**
**14C**	41 (20.5%)	32 (16%)	34 (17%)	**62 (31%)**	31 (15.5%)
**14D**	**101 (50.5%)**	54 (27%)	19 (9.5%)	17 (8.5%)	9 (4.5%)
**14E**	38 (19%)	**68 (34%)**	53 (26.5%)	33 (16.5%)	8 (4%)
**15A**	31 (15.5%)	**85 (42.5%)**	54 (27%)	17 (8.5%)	13 (6.5%)
**15B**	24 (12%)	21 (10.5%)	52 (26%)	**56 (28%)**	47 (23.5%)
**15C**	**73 (36.5%)**	40 (20%)	30 (15%)	33 (16.5%)	24 (12%)
**15D**	24 (12%)	23 (11.5%)	35 (17.5%)	31 (15.5%)	**87 (43.5%)**
**15E**	48 (24%)	31 (15.5%)	29 (14.5%)	**63 (31.5%)**	29 (14.5%)
**16A**	27 (13.5%)	35 (17.5%)	**63 (31.5%)**	50 (25%)	25 (12.5%)

**16B**	2 (1%)	4 (2%)	10 (5%)	48 (24%)	**136 (68%)**
**16C**	39 (19.5%)	29 (14.5%)	38 (19%)	**80 (40%)**	14 (7%)
**16D**	**88 (44%)**	84 (42%)	18 (9%)	6 (3%)	4 (2%)
**16E**	44 (22%)	48 (24%)	**71 (35.5%)**	16 (8%)	21 (10.5%)
**17A**	8 (4%)	**73 (36.5%)**	63 (31.5%)	46 (23%)	10 (5%)
**17B**	33 (16.5%)	21 (10.5%)	31 (15.5%)	**60 (30%)**	55 (27.5%)
**17C**	12 (6%)	37 (18.5%)	47 (23.5%)	**53 (26.5%)**	51 (25.5%)
**17D**	**88 (44%)**	42 (21%)	26 (13%)	22 (11%)	22 (11%)
**17E**	59 (29.5%)	27 (13.5%)	33 (16.5%)	19 (9.5%)	**62 (31%)**
**18A**	41 (20.5%)	**57 (28.5%)**	55 (27.5%)	23 (11.5%)	24 (12%)
**18B**	25 (12.5%)	25 (12.5%)	52 (26%)	**58 (29%)**	40 (20%)
**18C**	9 (4.5%)	20 (10%)	32 (16%)	50 (25%)	**89 (44.5%)**
**18D**	36 (18%)	53 (26.5%)	27 (13.5%)	**58 (29%)**	26 (13%)
**18E**	**89 (44.5%)**	45 (22.5%)	34 (17%)	11 (5.5%)	21 (10.5%)
**19A**	29 (14.5%)	**69 (34.5%)**	62 (31%)	39 (19.5%)	1 (0.5%)
**19B**	1 (0.5%)	5 (2.5%)	40 (20%)	38 (19%)	**116 (58%)**
**19C**	2 (1%)	9 (4.5%)	19 (9.5%)	**103 (51.5%)**	67 (33.5%)
**19D**	**87 (43.5%)**	54 (27%)	33 (16.5%)	18 (9%)	8 (4%)
**19E**	**81 (40.5%)**	63 (31.5%)	46 (23%)	2 (1%)	8 (4%)
**20A**	7 (3.5%)	41 (20.5%)	**66 (33%)**	61 (30.5%)	25 (12.5%)
**20B**	5 (2.5%)	8 (4%)	30 (15%)	26 (13%)	**131 (65.5%)**
**20C**	40 (20%)	46 (23%)	36 (18%)	**69 (34.5%)**	9 (4.5%)
**20D**	**67 (33.5%)**	49 (24.5%)	35 (17.5%)	36 (18%)	13 (6.5%)
**20E**	**81 (40.5%)**	56 (28%)	33 (16.5%)	8 (4%)	22 (11%)
**21A**	22 (11%)	37 (18.5%)	20 (10%)	49 (24.5%)	**72 (36%)**
**21B**	17 (8.5%)	34 (17%)	23 (11.5%)	48 (24%)	**78 (39%)**
**21C**	49 (24.5%)	37 (18.5%)	**50 (25%)**	**50 (25%)**	14 (7%)
**21D**	**59 (29.5%)**	70 (35%)	56 (28%)	12 (6%)	3 (1.5%)
**21E**	**53 (26.5%)**	22 (11%)	51 (25.5%)	41 (20.5%)	33 (16.5%)
**22A**	49 (24.5%)	**73 (36.5%)**	40 (20%)	30 (15%)	8 (4%)
**22B**	17 (8.5%)	27 (13.5%)	6 (3%)	69 (34.5%)	**81 (40.5%)**
**22C**	**83 (41.5%)**	49 (24.5%)	21 (10.5%)	40 (20%)	7 (3.5%)
**22D**	15 (7.5%)	23 (11.5%)	**73 (36.5%)**	47 (23.5%)	42 (21%)
**22E**	36 (18%)	28 (14%)	60 (30%)	14 (7%)	**62 (31%)**

Table S2. Construction of subdomains scores analyzed in the study

Score - Appointment (Cronbach alpha = 0.676, p<0.001, λ = 2.567)*		
Item	*PCA component coefficient*	*Alpha – Item deleted*
**8A**	0.619	0.631
**8B**	0.486	0.653
**8D**	-0.458	0.670
**8E**	-0.529	0.661
**9A**	0.574	0.642
**9B**	0.386	0.673
**9D**	-0.605	0.633
**10C**	0.521	0.648
**10D**	-0.584	0.636
Score – Diagnosis (Cronbach alpha = 0.704, p<0.001, λ = 2.632)*		
**11C**	-0.508	0.684
**11E**	0.476	0.692
**12B**	0.577	0.674
**12C**	-0.754	0.637
**12D**	-0.477	0.696
**12E**	0.696	0.640
**13A**	0.525	0.685
**13E**	-0.508	0.685
Score - Treatment (Cronbach alpha = 0.701, p<0.001, λ = 2.831)*		
**14B**	0.483	0.682
**14C**	0.481	0.685
**14D**	-0.496	0.680
**14E**	-0.372	0.694
**15A**	-0.335	0.696
**15C**	-0.570	0.672
**15D**	0.517	0.681
**15E**	0.501	0.681
**16A**	0.452	0.691
**16C**	-0.761	0.639
**16E**	0.493	0.684
Score - Feedback (Cronbach alpha = 0.770, p<0.001, λ = 3.229)*		
**17C**	-0.683	0.733
**17E**	0.686	0.738
**18A**	0.548	0.754
**18B**	0.716	0.728
**18C**	-0.511	0.760
**18D**	-0.691	0.732
**19B**	0.397	0.770
**19C**	-0.577	0.753
**19E**	0.500	0.761
Score – Dispensarization (Cronbach alpha = 0.745, p<0.001, λ = 3.144)*		

**20B**	0.319	0.743
**20C**	0.541	0.725
**20D**	-0.670	0.708
**21B**	0.766	0.687
**21C**	0.577	0.725
**21D**	-0.496	0.732
**21E**	-0.573	0.728
**22B**	0.341	0.749
**22D**	0.388	0.747
**22E**	-0.735	0.689

***Bartlett's Test of Sphericity**

Table S3. Construction of interest scores regarding digitalization

Score - Pragmatism (Cronbach alpha = 0.907, p<0.001*, λ = 8.638)		
Item	***PCA component coefficient***	***Alpha – Item deleted***
**8C**	0.481	0.905
**8D**	-0.329	0.908
**8E**	-0.306	0.908
**9C**	-0.452	0.906
**10C**	0.559	0.904
**10D**	-0.385	0.907
**11C**	-0.307	0.907
**12B**	0.488	0.905
**12C**	-0.792	0.899
**12D**	-0.310	0.908
**12E**	0.697	0.901
**14A**	-0.439	0.906
**14B**	0.499	0.905
**14C**	0.443	0.906
**14E**	-0.370	0.907
**16A**	0.467	0.905
**16C**	-0.717	0.901
**17C**	-0.633	0.903
**17E**	0.644	0.902
**18A**	0.347	0.907
**18B**	0.653	0.902
**18D**	-0.614	0.903
**19C**	-0.442	0.906
**19E**	0.501	0.905
**20C**	-0.430	0.906
**20D**	0.504	0.905
**21A**	0.581	0.903
**21B**	-0.708	0.901

**21C**	-0.670	0.902
**21D**	0.457	0.906
**22E**	0.610	0.903
Score – Efficiency vs. human impact (Cronbach alpha = 0.850, p<0.001*, λ = 5.475)		
**8A**	0.379	0.848
**8B**	0.338	0.849
**9B**	0.526	0.843
**9D**	-0.369	0.849
**9E**	-0.365	0.852
**13A**	-0.589	0.842
**13C**	0.392	0.848
**13D**	0.660	0.836
**13E**	-0.321	0.850
**16B**	0.439	0.847
**16E**	-0.504	0.844
**17B**	0.689	0.834
**17D**	-0.780	0.829
**18C**	0.553	0.843
**19D**	-0.439	0.846
**20B**	0.619	0.841
**20E**	-0.563	0.841
**21E**	0.372	0.850
**22A**	-0.598	0.841
**22B**	0.638	0.840

***Bartlett's Test of Sphericity**

Appointment score construction

Descriptive Statistics

	Mean	Std. Deviation	Analysis N
8A	2.92	1.100	195
8B	4.24	1.020	195
8C	2.25	1.228	195
8D	2.85	1.409	195
8E	2.74	1.449	195
9A	3.07	1.290	195
9B	4.05	1.159	195
9C	2.75	1.325	195
9D	2.32	1.273	195
9E	2.82	1.413	195
10A	2.67	1.092	195
10B	4.51	0.864	195
10C	2.28	1.216	195
10D	2.76	1.364	195
10E	2.79	1.325	195

Communalities

	Initial	Extraction
8A	1.000	0.511
8B	1.000	0.692
8C	1.000	0.748
8D	1.000	0.826
8E	1.000	0.889

9A	1.000	0.713
9B	1.000	0.704
9C	1.000	0.765
9D	1.000	0.684
9E	1.000	0.893
10A	1.000	0.898
10B	1.000	0.562
10C	1.000	0.695
10D	1.000	0.829
10E	1.000	0.929

Extraction Method: Principal Component Analysis.

Total Variance Explained

Component	Initial Eigenvalues			Extraction Sums of Squared Loadings			Rotation Sums of Squared Loadings		
	Total	% of Variance	Cumulative %	Total	% of Variance	Cumulative %	Total	% of Variance	Cumulative %
1	2.746	18.305	18.305	2.746	18.305	18.305	2.131	14.205	14.205
2	1.955	13.032	31.337	1.955	13.032	31.337	1.979	13.194	27.400
3	1.600	10.666	42.003	1.600	10.666	42.003	1.637	10.915	38.314
4	1.473	9.821	51.824	1.473	9.821	51.824	1.482	9.882	48.196
5	1.375	9.168	60.992	1.375	9.168	60.992	1.463	9.752	57.949
6	1.101	7.340	68.332	1.101	7.340	68.332	1.363	9.088	67.037
7	1.089	7.258	75.590	1.089	7.258	75.590	1.283	8.553	75.590
8	0.875	5.831	81.421						
9	0.803	5.355	86.776						
10	0.787	5.248	92.023						

11	0.640	4.267	96.290						
12	0.555	3.701	99.991						
13	0.001	0.009	100.000						
14	7.586E-16	5.057E-15	100.000						
15	-4.706E-16	-3.137E-15	100.000						

Extraction Method: Principal Component Analysis.

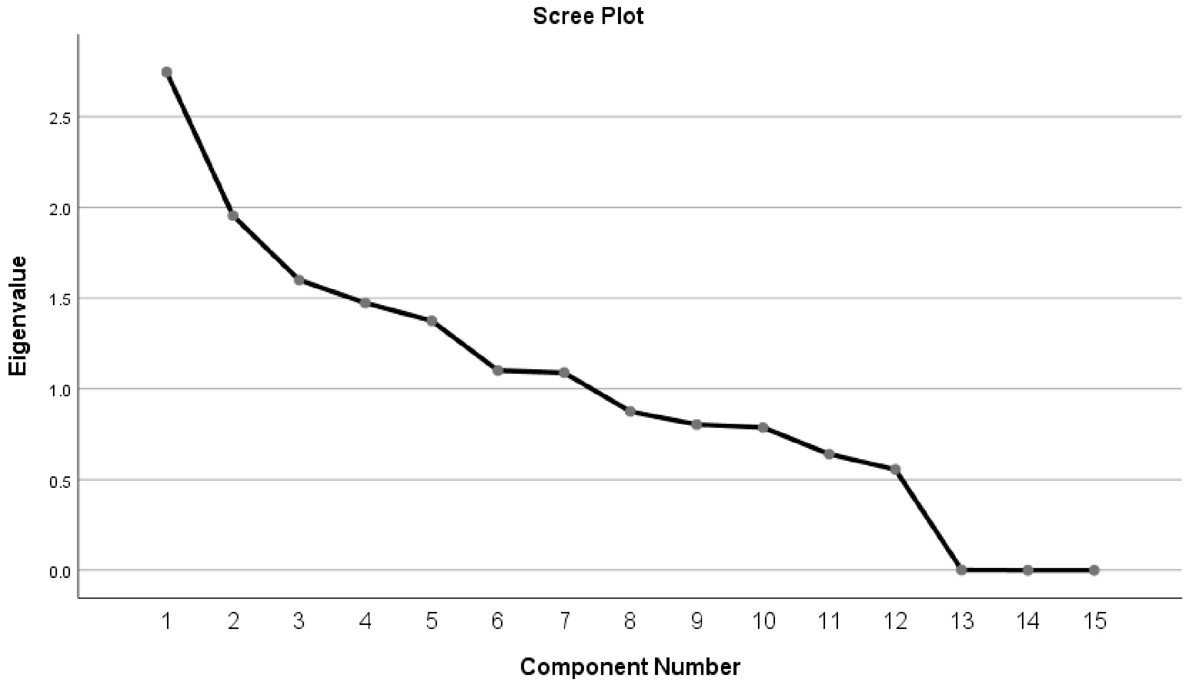

Component Matrix[a]

	Component						
	1	2	3	4	5	6	7
8A	0.573	0.333					
8B	0.417					0.429	0.457
8C	0.321	-0.687					

8D	-0.507			-0.368	-0.514		
8E	-0.508			0.433	0.484	-0.325	
9A	0.652	-0.354				0.302	
9B	0.338	0.678					
9C	-0.320		0.677				-0.372
9D	-0.528	-0.336			0.333	0.366	
9E			-0.688	-0.415	-0.312		
10A			0.589			-0.428	0.533
10B		0.581	-0.323				
10C	0.572	-0.452					-0.324
10D	-0.560			0.427	-0.455		
10E				-0.803	0.505		

Extraction Method: Principal Component Analysis.

a. 7 components extracted.

Final model

Descriptive Statistics

	Mean	Std. Deviation	Analysis N
8A	2.92	1.105	198
8B	4.25	1.016	198
8D	2.83	1.407	198
8E	2.74	1.443	198
9A	3.06	1.291	198
9B	4.05	1.152	198
9D	2.31	1.271	198
10C	2.27	1.212	198

10D	2.74	1.367	198

KMO and Bartlett's Test

Kaiser-Meyer-Olkin Measure of Sampling Adequacy.		0.435
Bartlett's Test of Sphericity	Approx. Chi-Square	414.957
	df	36
	Sig.	0.000

Communalities

	Initial	Extraction
8A	1.000	0.425
8B	1.000	0.252
8D	1.000	0.812
8E	1.000	0.848
9A	1.000	0.651
9B	1.000	0.786
9D	1.000	0.572
10C	1.000	0.525
10D	1.000	0.382

Extraction Method: Principal Component Analysis.

Total Variance Explained

ComponentInitial Eigenvalues	Extraction Sums of Squared Loadings	Rotation Sums of Squared Loadings

	Total	% of Variance	Cumulative %	Total	% of Variance	Cumulative %	Total	% of Variance	Cumulative %
1	2.567	28.519	28.519	2.567	28.519	28.519	1.927	21.409	21.409
2	1.459	16.208	44.727	1.459	16.208	44.727	1.712	19.019	40.427
3	1.229	13.655	58.382	1.229	13.655	58.382	1.616	17.955	58.382
4	0.968	10.757	69.140						
5	0.879	9.762	78.902						
6	0.773	8.586	87.488						
7	0.557	6.185	93.673						
8	0.393	4.368	98.041						
9	0.176	1.959	100.000						

Extraction Method: Principal Component Analysis.

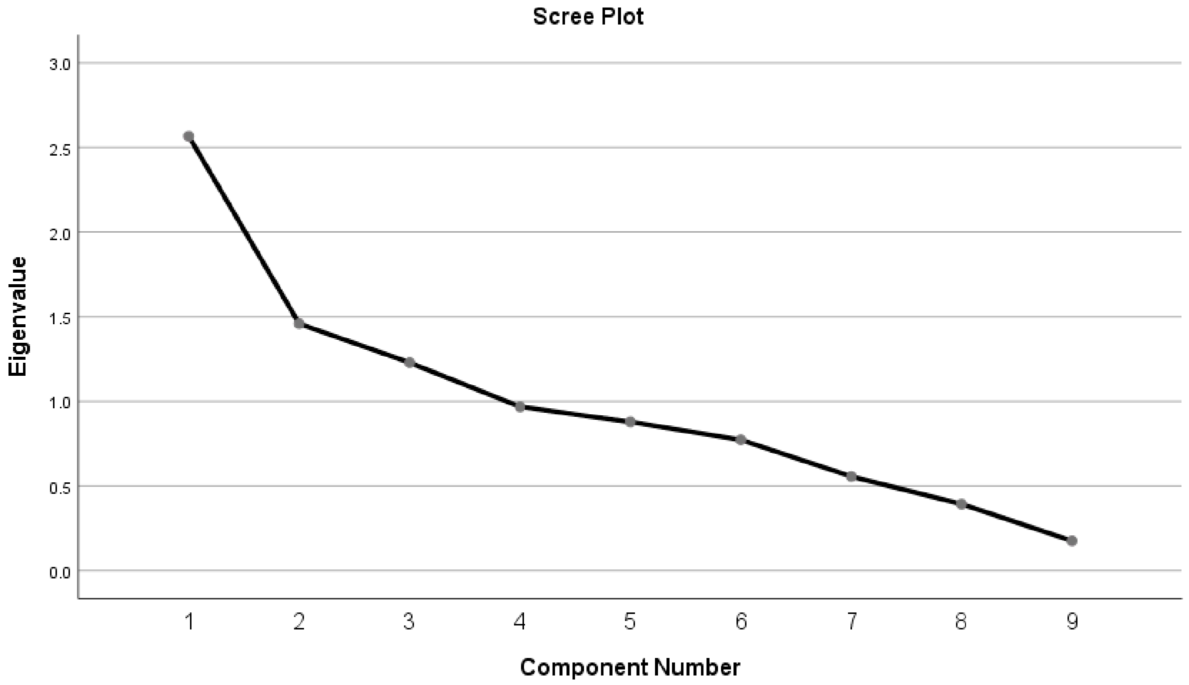

Component Matrix[a]

	Component		
	1	2	3
8A	0.619		
8B	0.486		
8D	-0.458	-0.622	0.464
8E	-0.529	0.466	-0.592
9A	0.574		0.483
9B	0.386	-0.543	-0.585
9D	-0.605	0.435	
10C	0.521	0.489	
10D	-0.584		

Extraction Method: Principal Component Analysis.

a. 3 components extracted.

Diagnosis score construction

Descriptive Statistics

	Mean	Std. Deviation	Analysis N
11A	2.83	1.226	200
11B	4.32	1.035	200
11C	1.88	1.180	200
11D	3.32	1.154	200
11E	2.67	1.229	200
12A	3.41	1.048	200
12B	3.09	1.133	200
12C	3.15	1.800	200

12D	2.97	1.333	200
12E	2.39	1.442	200
13A	2.10	1.084	200
13B	3.14	1.045	200
13C	4.61	0.789	200
13D	3.04	1.291	200
13E	2.12	1.137	200

Communalities

	Initial	Extraction
11A	1.000	0.780
11B	1.000	0.746
11C	1.000	0.776
11D	1.000	0.754
11E	1.000	0.909
12A	1.000	0.780
12B	1.000	0.607
12C	1.000	0.725
12D	1.000	0.684
12E	1.000	0.672
13A	1.000	0.692
13B	1.000	0.831
13C	1.000	0.757
13D	1.000	0.860
13E	1.000	0.798

Extraction Method: Principal Component Analysis.

Total Variance Explained

Component	Initial Eigenvalues			Extraction Sums of Squared Loadings			Rotation Sums of Squared Loadings		
	Total	% of Variance	Cumulative %	Total	% of Variance	Cumulative %	Total	% of Variance	Cumulative %
1	2.845	18.967	18.967	2.845	18.967	18.967	2.081	13.877	13.877
2	2.265	15.098	34.065	2.265	15.098	34.065	1.919	12.793	26.669
3	1.550	10.332	44.397	1.550	10.332	44.397	1.664	11.094	37.764
4	1.337	8.910	53.307	1.337	8.910	53.307	1.532	10.213	47.977
5	1.255	8.369	61.676	1.255	8.369	61.676	1.443	9.620	57.597
6	1.064	7.094	68.770	1.064	7.094	68.770	1.378	9.184	66.781
7	1.054	7.026	75.796	1.054	7.026	75.796	1.352	9.014	75.796
8	0.897	5.982	81.777						
9	0.814	5.424	87.202						
10	0.724	4.825	92.027						
11	0.640	4.266	96.293						
12	0.556	3.707	100.000						
13	8.829E-16	5.886E-15	100.000						
14	-3.193E-16	-2.129E-15	100.000						
15	-1.357E-15	-9.049E-15	100.000						

Extraction Method: Principal Component Analysis.

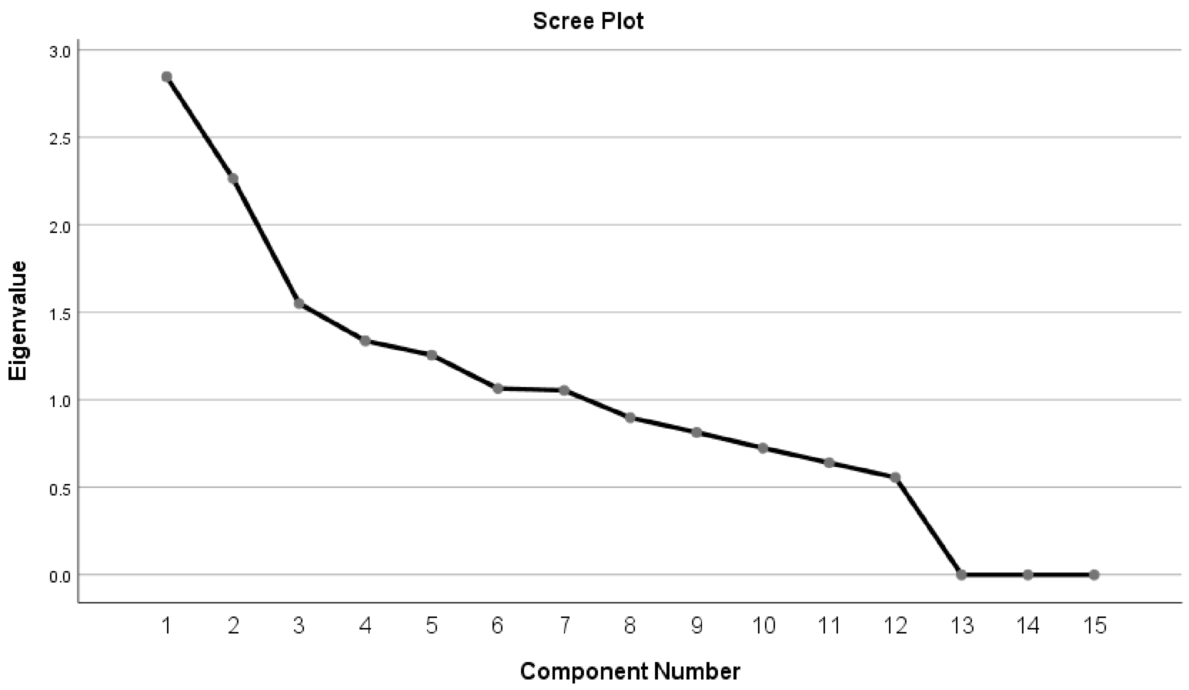

Component Matrix[a]

	Component						
	1	2	3	4	5	6	7
11A			-0.644		-0.521		
11B		-0.401	0.530	0.361			
11C	-0.516		-0.350	0.322	0.300	-0.389	
11D		0.436	0.428		-0.536		
11E	0.439			-0.567	0.495		
12A	0.451		0.321			-0.505	0.407
12B	0.511					0.381	
12C	-0.733	-0.417					
12D	-0.520			-0.492			

12E	0.667						
13A	0.480	0.588					
13B		0.399	0.394	0.556			-0.325
13C		-0.630				0.327	0.426
13D		-0.719				-0.301	-0.379
13E	-0.547	0.325	-0.354		0.320		0.381

Extraction Method: Principal Component Analysis.

a. 7 components extracted.

Final model

Descriptive Statistics

	Mean	Std. Deviation	Analysis N
11C	1.88	1.180	200
11E	2.67	1.229	200
12B	3.09	1.133	200
12C	3.15	1.800	200
12D	2.97	1.333	200
12E	2.39	1.442	200
13A	2.10	1.084	200
13E	2.12	1.137	200

KMO and Bartlett's Test

Kaiser-Meyer-Olkin Measure of Sampling Adequacy.		0.392
Bartlett's Test of Sphericity	Approx. Chi-Square	455.874
	df	28

Sig.	0.000

Communalities

	Initial	Extraction
11C	1.000	0.690
11E	1.000	0.587
12B	1.000	0.525
12C	1.000	0.792
12D	1.000	0.864
12E	1.000	0.572
13A	1.000	0.602
13E	1.000	0.431

Extraction Method: Principal Component Analysis.

Total Variance Explained

	Initial Eigenvalues			Extraction Sums of Squared Loadings			Rotation Sums of Squared Loadings		
Component	Total	% of Variance	Cumulative %	Total	% of Variance	Cumulative %	Total	% of Variance	Cumulative %
1	2.632	32.900	32.900	2.632	32.900	32.900	1.818	22.723	22.723
2	1.246	15.578	48.478	1.246	15.578	48.478	1.625	20.313	43.036
3	1.185	14.807	63.285	1.185	14.807	63.285	1.620	20.249	63.285
4	0.893	11.168	74.453						
5	0.793	9.910	84.363						
6	0.649	8.115	92.479						
7	0.491	6.137	98.616						

8	0.111	1.384	100.000						

Extraction Method: Principal Component Analysis.

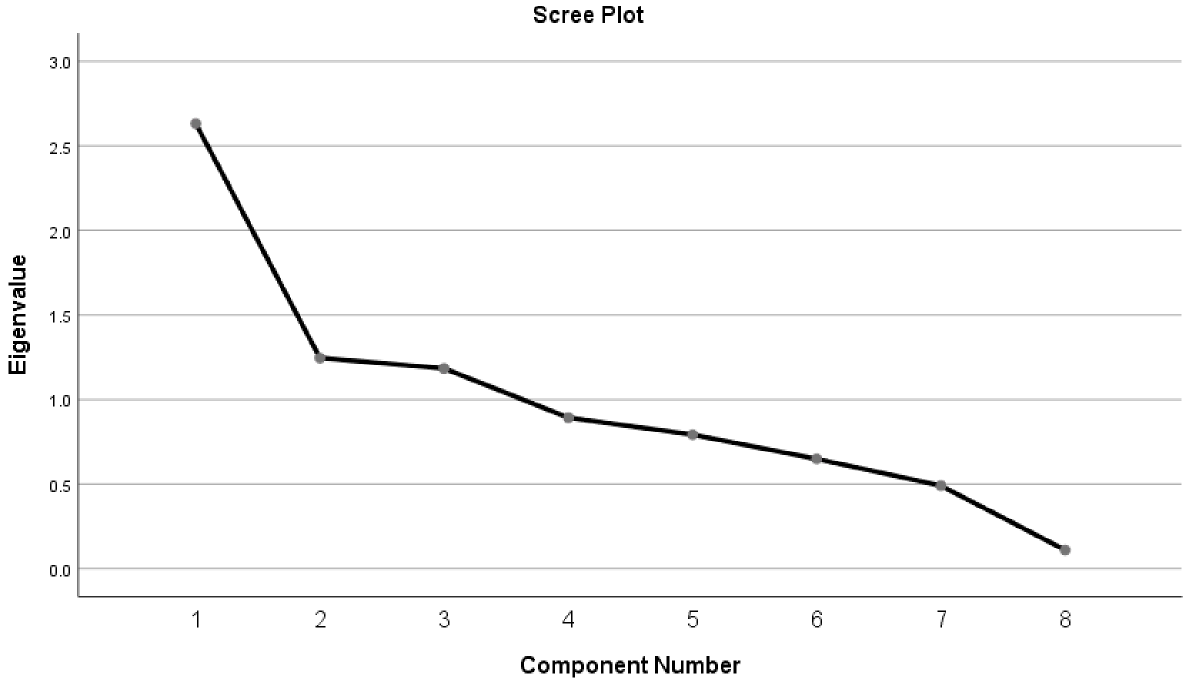

Component Matrix[a]

	Component		
	1	2	3
11C	-0.508	0.590	
11E	0.476	-0.599	
12B	0.577	0.407	
12C	-0.754		-0.471
12D	-0.477	-0.515	0.610
12E	0.696		
13A	0.525		0.571
13E	-0.508		0.361

Extraction Method: Principal Component Analysis.

a. 3 components extracted.

Treatment score construction

Descriptive Statistics

	Mean	Std. Deviation	Analysis N
14A	3.61	1.133	200
14B	3.92	1.273	200
14C	3.05	1.385	200
14D	1.90	1.158	200
14E	2.53	1.098	200
15A	2.48	1.061	200
15B	3.41	1.284	200
15C	2.48	1.428	200
15D	3.67	1.432	200
15E	2.97	1.421	200
16A	3.06	1.212	200
16B	4.56	0.768	200
16C	3.01	1.270	200
16D	1.77	0.884	200
16E	2.61	1.215	200

Communalities

	Initial	Extraction
14A	1.000	0.603

14B	1.000	0.778
14C	1.000	0.763
14D	1.000	0.724
14E	1.000	0.471
15A	1.000	0.760
15B	1.000	0.706
15C	1.000	0.793
15D	1.000	0.670
15E	1.000	0.576
16A	1.000	0.826
16B	1.000	0.640
16C	1.000	0.706
16D	1.000	0.673
16E	1.000	0.833

Extraction Method: Principal Component Analysis.

Total Variance Explained

	Initial Eigenvalues			Extraction Sums of Squared Loadings			Rotation Sums of Squared Loadings		
Component	Total	% of Variance	Cumulative %	Total	% of Variance	Cumulative %	Total	% of Variance	Cumulative %
1	3.046	20.306	20.306	3.046	20.306	20.306	2.467	16.448	16.448
2	2.189	14.595	34.901	2.189	14.595	34.901	2.200	14.670	31.118
3	1.709	11.394	46.295	1.709	11.394	46.295	1.650	11.001	42.119
4	1.308	8.717	55.011	1.308	8.717	55.011	1.583	10.556	52.675
5	1.187	7.914	62.926	1.187	7.914	62.926	1.399	9.326	62.001

6	1.084	7.225	70.151	1.084	7.225	70.151	1.223	8.150	70.151
7	0.996	6.637	76.788						
8	0.873	5.821	82.609						
9	0.738	4.920	87.529						
10	0.710	4.734	92.264						
11	0.611	4.070	96.334						
12	0.550	3.666	100.000						
13	7.196E-16	4.797E-15	100.000						
14	-1.065E-15	-7.102E-15	100.000						
15	-1.478E-15	-9.853E-15	100.000						

Extraction Method: Principal Component Analysis.

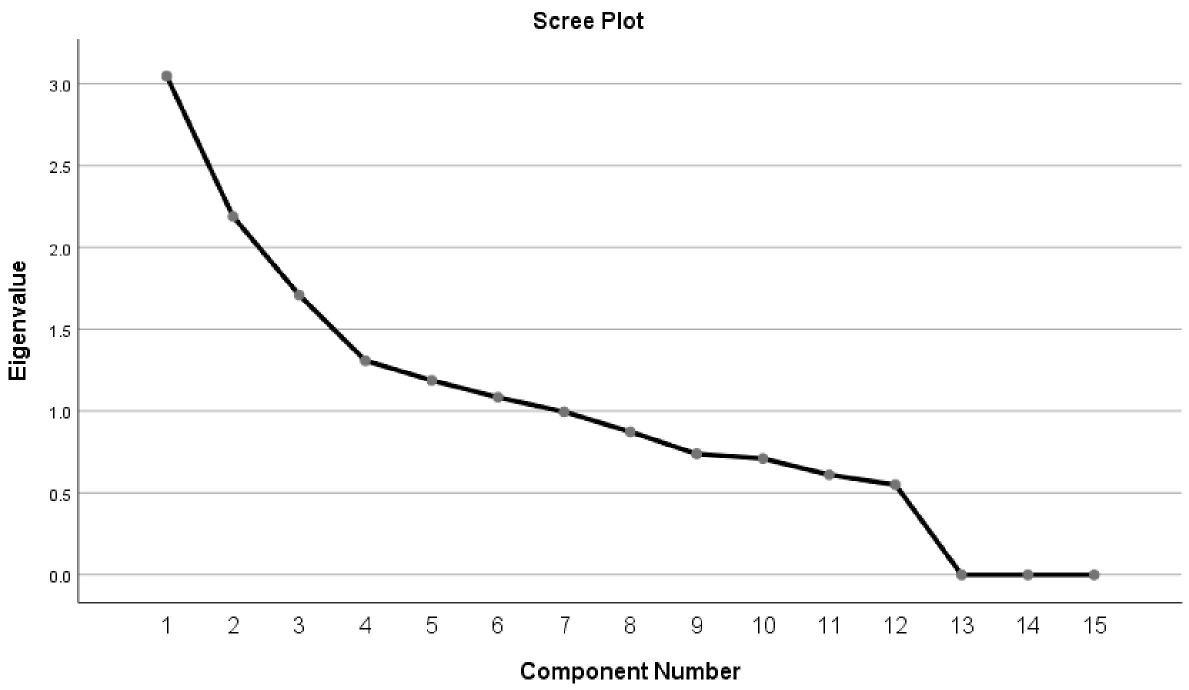

Component Matrix[a]

	Component					
	1	2	3	4	5	6
14A	0.384	0.650				
14B	-0.564	-0.455			-0.417	
14C	-0.501	-0.582			0.342	
14D	0.509		0.369			-0.506
14E	0.352	0.312	-0.359			0.340
15A	0.314					0.682
15B		-0.538			0.502	
15C	0.489	-0.461	-0.346		-0.335	
15D	-0.484	0.476				
15E	-0.496	0.446				
16A	-0.448	0.300	-0.304	0.659		
16B			-0.567		0.478	
16C	0.720					
16D			0.661	0.437		
16E	-0.512		0.336	-0.619		

Extraction Method: Principal Component Analysis.

a. 6 components extracted.

Final model

Descriptive Statistics

	Mean	Std. Deviation	Analysis N
14B	3.92	1.273	200

14C	3.05	1.385	200
14D	1.90	1.158	200
14E	2.53	1.098	200
15A	2.48	1.061	200
15C	2.48	1.428	200
15D	3.67	1.432	200
15E	2.97	1.421	200
16A	3.06	1.212	200
16C	3.01	1.270	200
16E	2.61	1.215	200

KMO and Bartlett's Test

Kaiser-Meyer-Olkin Measure of Sampling Adequacy.		0.370
Bartlett's Test of Sphericity	Approx. Chi-Square	774.716
	df	55
	Sig.	0.000

Communalities

	Initial	Extraction
14B	1.000	0.647
14C	1.000	0.639
14D	1.000	0.859
14E	1.000	0.809
15A	1.000	0.860
15C	1.000	0.821

15D	1.000	0.630
15E	1.000	0.522
16A	1.000	0.923
16C	1.000	0.676
16E	1.000	0.745

Extraction Method: Principal Component Analysis.

Total Variance Explained

	Initial Eigenvalues			Extraction Sums of Squared Loadings			Rotation Sums of Squared Loadings		
Component	Total	% of Variance	Cumulative %	Total	% of Variance	Cumulative %	Total	% of Variance	Cumulative %
1	2.831	25.735	25.735	2.831	25.735	25.735	2.241	20.373	20.373
2	1.700	15.450	41.185	1.700	15.450	41.185	1.781	16.193	36.566
3	1.390	12.638	53.823	1.390	12.638	53.823	1.471	13.369	49.935
4	1.134	10.305	64.128	1.134	10.305	64.128	1.360	12.360	62.295
5	1.076	9.785	73.912	1.076	9.785	73.912	1.278	11.617	73.912
6	0.891	8.098	82.010						
7	0.838	7.614	89.624						
8	0.628	5.707	95.331						
9	0.203	1.844	97.175						
10	0.166	1.508	98.683						
11	0.145	1.317	100.000						

Extraction Method: Principal Component Analysis.

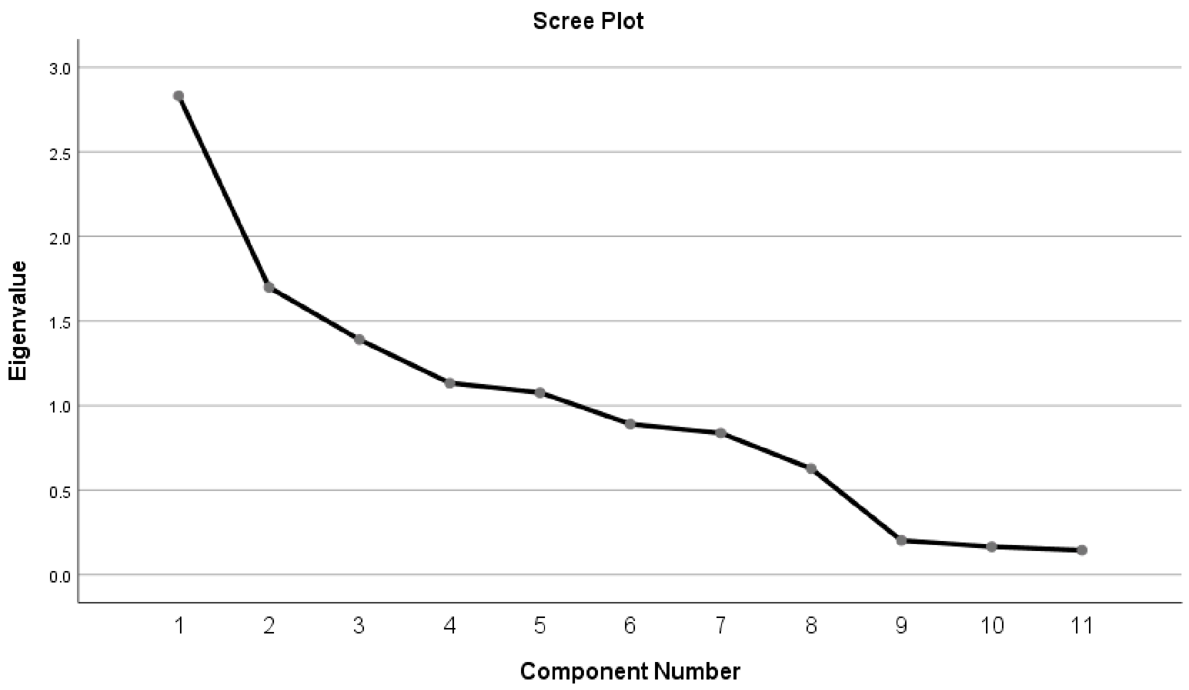

Component Matrix[a]

	Component				
	1	2	3	4	5
14B	0.483	0.551			
14C	0.481	0.562			
14D	-0.496	-0.393	-0.397		-0.498
14E	-0.372	-0.463	0.361	0.366	0.439
15A	-0.335			-0.657	0.526
15C	-0.570	0.520	0.342		
15D	0.517	-0.436		0.381	
15E	0.501	-0.388			
16A	0.452		0.680	-0.426	

16C	-0.761				
16E	0.493		-0.633		

Extraction Method: Principal Component Analysis.

a. 5 components extracted.

Feedback score construction

Descriptive Statistics

	Mean	Std. Deviation	Analysis N
17A	2.89	0.973	200
17B	3.42	1.415	200
17C	3.47	1.223	200
17D	2.24	1.397	200
17E	2.99	1.632	200
18A	2.66	1.262	200
18B	3.32	1.274	200
18C	3.95	1.189	200
18D	2.93	1.341	200
18E	2.15	1.329	200
19A	2.57	0.980	200
19B	4.32	0.911	200
19C	4.12	0.830	200
19D	2.03	1.151	200
19E	1.97	1.019	200

Communalities

	Initial	Extraction
17A	1.000	0.862
17B	1.000	0.685
17C	1.000	0.674
17D	1.000	0.750
17E	1.000	0.635
18A	1.000	0.602
18B	1.000	0.645
18C	1.000	0.763
18D	1.000	0.637
18E	1.000	0.799
19A	1.000	0.880
19B	1.000	0.768
19C	1.000	0.656
19D	1.000	0.823
19E	1.000	0.723

Extraction Method: Principal Component Analysis.

Total Variance Explained

	Initial Eigenvalues			Extraction Sums of Squared Loadings			Rotation Sums of Squared Loadings		
Component	Total	% of Variance	Cumulative %	Total	% of Variance	Cumulative %	Total	% of Variance	Cumulative %
1	3.388	22.587	22.587	3.388	22.587	22.587	2.688	17.922	17.922
2	2.396	15.975	38.562	2.396	15.975	38.562	2.231	14.875	32.796
3	1.607	10.715	49.277	1.607	10.715	49.277	1.735	11.566	44.362

4	1.286	8.575	57.852	1.286	8.575	57.852	1.555	10.369	54.732
5	1.164	7.757	65.609	1.164	7.757	65.609	1.498	9.989	64.721
6	1.060	7.070	72.678	1.060	7.070	72.678	1.194	7.958	72.678
7	0.966	6.441	79.119						
8	0.767	5.115	84.235						
9	0.735	4.902	89.137						
10	0.670	4.467	93.603						
11	0.563	3.751	97.354						
12	0.397	2.646	100.000						
13	-2.594E-16	-1.729E-15	100.000						
14	-7.219E-16	-4.813E-15	100.000						
15	-1.985E-15	-1.323E-14	100.000						

Extraction Method: Principal Component Analysis.

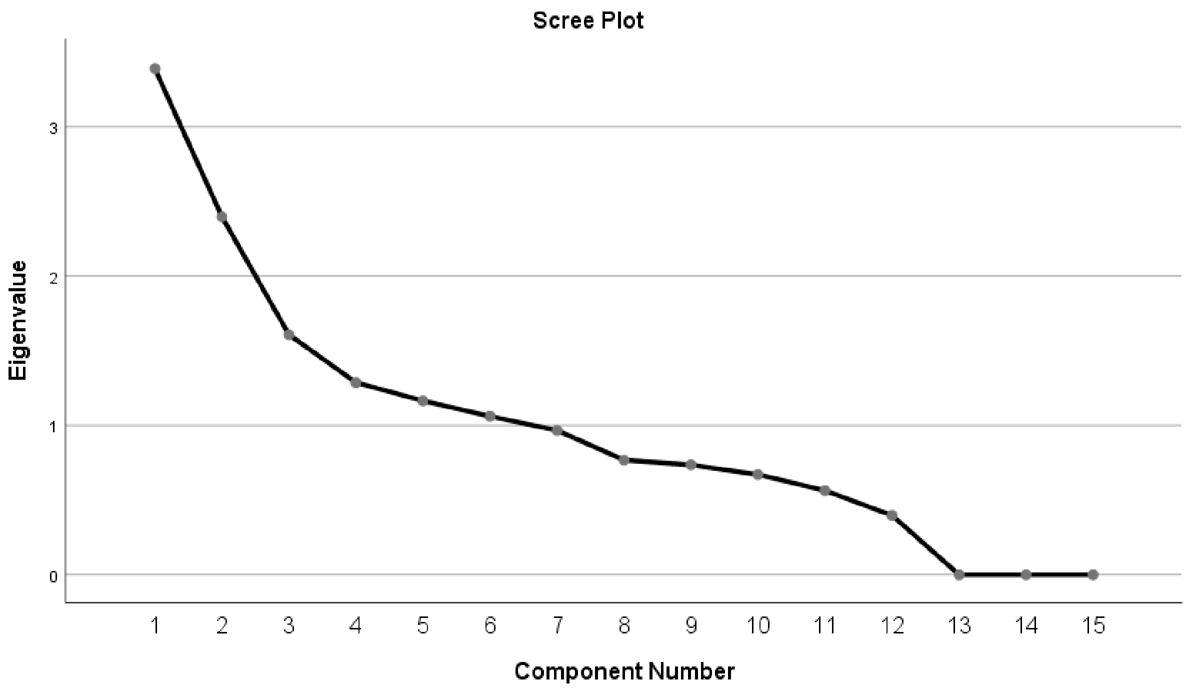

Component Matrix[a]

	Component					
	1	2	3	4	5	6
17A				0.430	0.584	-0.473
17B		0.765				
17C	0.631	-0.312				0.314
17D		-0.764				
17E	-0.673	0.302				
18A	-0.553			-0.392		
18B	-0.732		0.321			
18C	0.521	0.407	0.468			
18D	0.672					
18E			-0.820			
19A		0.351	-0.427	-0.415	0.539	
19B	-0.432		0.325	0.494		0.428
19C	0.544		0.329		-0.390	
19D		-0.753				-0.414
19E	-0.513			0.424	-0.394	

Extraction Method: Principal Component Analysis.

a. 6 components extracted.

Final model

Descriptive Statistics

	Mean	Std. Deviation	Analysis N

17C	3.47	1.223	200
17E	2.99	1.632	200
18A	2.66	1.262	200
18B	3.32	1.274	200
18C	3.95	1.189	200
18D	2.93	1.341	200
19B	4.32	0.911	200
19C	4.12	0.830	200
19E	1.97	1.019	200

KMO and Bartlett's Test

Kaiser-Meyer-Olkin Measure of Sampling Adequacy.		0.654
Bartlett's Test of Sphericity	Approx. Chi-Square	494.639
	df	36
	Sig.	0.000

Communalities

	Initial	Extraction
17C	1.000	0.639
17E	1.000	0.706
18A	1.000	0.787
18B	1.000	0.543
18C	1.000	0.719
18D	1.000	0.738
19B	1.000	0.209

19C	1.000	0.726
19E	1.000	0.485

Extraction Method: Principal Component Analysis.

Total Variance Explained

	Initial Eigenvalues			Extraction Sums of Squared Loadings			Rotation Sums of Squared Loadings		
Component	Total	% of Variance	Cumulative %	Total	% of Variance	Cumulative %	Total	% of Variance	Cumulative %
1	3.229	35.879	35.879	3.229	35.879	35.879	2.240	24.885	24.885
2	1.233	13.704	49.583	1.233	13.704	49.583	1.748	19.424	44.309
3	1.089	12.097	61.680	1.089	12.097	61.680	1.563	17.371	61.680
4	0.984	10.933	72.613						
5	0.807	8.963	81.576						
6	0.596	6.628	88.204						
7	0.497	5.523	93.727						
8	0.327	3.631	97.357						
9	0.238	2.643	100.000						

Extraction Method: Principal Component Analysis.

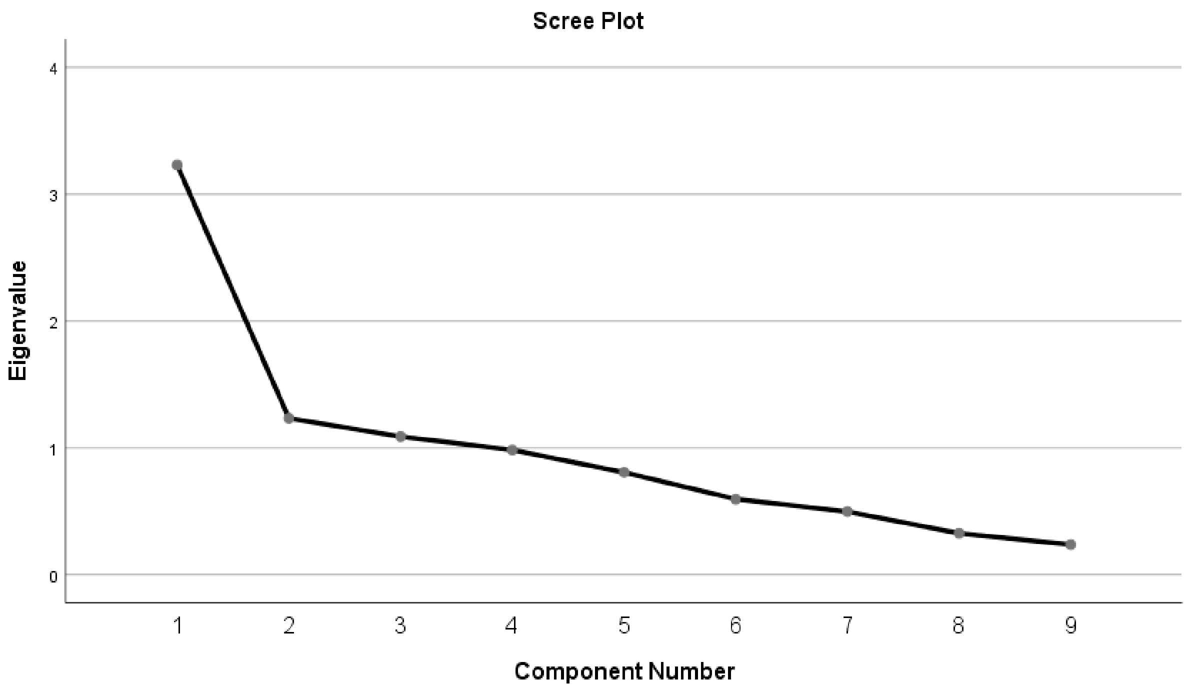

Component Matrix[a]

	Component		
	1	2	3
17C	-0.683	-0.395	
17E	0.686	0.450	
18A	0.548	-0.303	-0.628
18B	0.716		
18C	-0.511	0.674	
18D	-0.691		0.431
19B	0.397		
19C	-0.577	0.472	-0.413
19E	0.500		0.485

Extraction Method: Principal Component Analysis.

a. 3 components extracted.

Dispensarization score construction

Descriptive Statistics

	Mean	Std. Deviation	Analysis N
20A	3.28	1.038	200
20B	4.35	1.036	200
20C	2.81	1.235	200
20D	2.40	1.291	200
20E	2.17	1.304	200
21A	3.56	1.416	200
21B	3.68	1.363	200
21C	2.72	1.274	200
21D	2.15	0.965	200
21E	2.90	1.426	200
22A	2.38	1.127	200
22B	3.85	1.314	200
22C	2.20	1.267	200
22D	3.39	1.160	200
22E	3.19	1.464	200

Communalities

	Initial	Extraction
20A	1.000	0.647

20B	1.000	0.807
20C	1.000	0.710
20D	1.000	0.722
20E	1.000	0.827
21A	1.000	0.865
21B	1.000	0.731
21C	1.000	0.751
21D	1.000	0.646
21E	1.000	0.758
22A	1.000	0.711
22B	1.000	0.784
22C	1.000	0.858
22D	1.000	0.599
22E	1.000	0.766

Extraction Method: Principal Component Analysis.

Total Variance Explained

	Initial Eigenvalues			Extraction Sums of Squared Loadings			Rotation Sums of Squared Loadings		
Component	Total	% of Variance	Cumulative %	Total	% of Variance	Cumulative %	Total	% of Variance	Cumulative %
1	3.441	22.941	22.941	3.441	22.941	22.941	2.403	16.022	16.022
2	2.652	17.681	40.623	2.652	17.681	40.623	2.282	15.211	31.233
3	1.698	11.322	51.944	1.698	11.322	51.944	1.786	11.910	43.143
4	1.248	8.317	60.261	1.248	8.317	60.261	1.651	11.010	54.152
5	1.095	7.298	67.559	1.095	7.298	67.559	1.541	10.275	64.427

6	1.048	6.988	74.547	1.048	6.988	74.547	1.518	10.120	74.547
7	0.924	6.158	80.705						
8	0.823	5.485	86.190						
9	0.724	4.829	91.019						
10	0.604	4.030	95.049						
11	0.432	2.877	97.926						
12	0.311	2.074	100.000						
13	5.661E-16	3.774E-15	100.000						
14	-4.820E-16	-3.213E-15	100.000						
15	-9.862E-16	-6.575E-15	100.000						

Extraction Method: Principal Component Analysis.

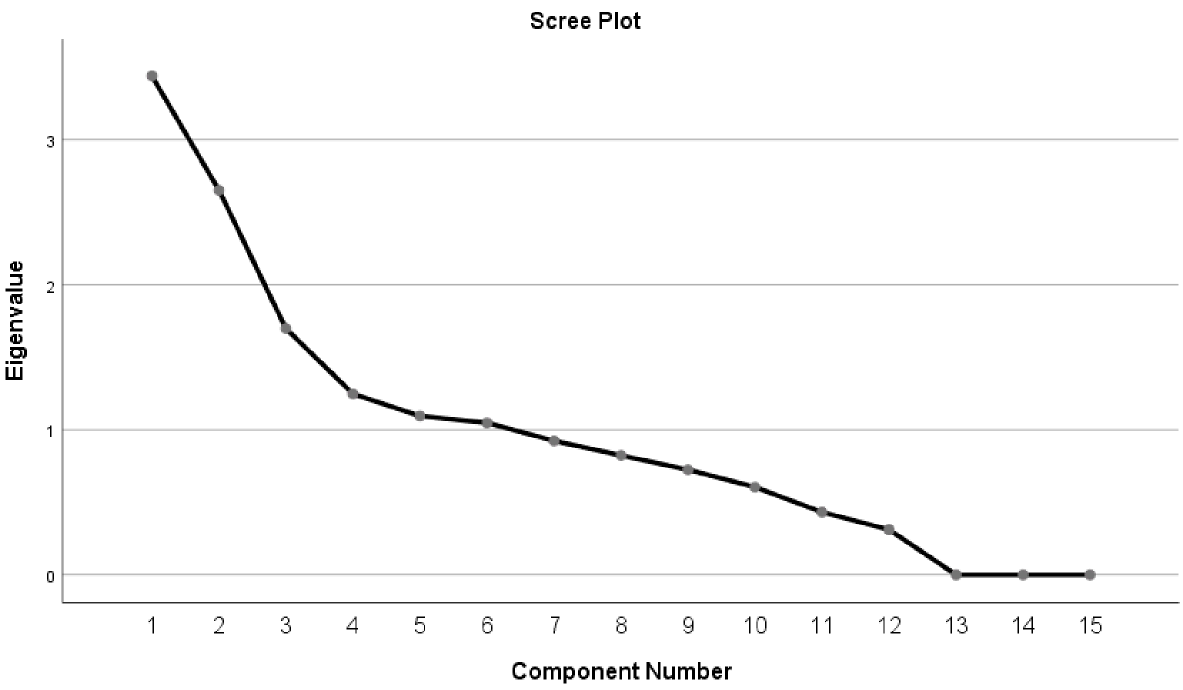

Component Matrix[a]

	Component					
	1	2	3	4	5	6
20A		0.538		-0.389	0.340	
20B	0.342	-0.753		0.305		
20C	0.542				0.394	
20D	-0.616				-0.363	0.360
20E		0.578	-0.407		-0.362	-0.410
21A	-0.441		0.620			-0.513
21B	0.772					
21C	0.599	0.446				0.310
21D	-0.510			0.422	0.377	
21E	-0.489	-0.384	-0.429	-0.363		
22A		0.473		0.525		0.390
22B	0.397	-0.787				
22C			0.688		-0.430	
22D	0.355		-0.433		0.408	
22E	-0.726		-0.329			

Extraction Method: Principal Component Analysis.

a. 6 components extracted.

Final model

Descriptive Statistics

	Mean	Std. Deviation	Analysis N
20B	4.35	1.036	200

20C	2.81	1.235	200
20D	2.40	1.291	200
21B	3.68	1.363	200
21C	2.72	1.274	200
21D	2.15	0.965	200
21E	2.90	1.426	200
22B	3.85	1.314	200
22D	3.39	1.160	200
22E	3.19	1.464	200

KMO and Bartlett's Test

Kaiser-Meyer-Olkin Measure of Sampling Adequacy.		0.614
Bartlett's Test of Sphericity	Approx. Chi-Square	620.167
	df	45
	Sig.	0.000

Communalities

	Initial	Extraction
20B	1.000	0.814
20C	1.000	0.828
20D	1.000	0.618
21B	1.000	0.675
21C	1.000	0.629
21D	1.000	0.897
21E	1.000	0.753

22B	1.000	0.817
22D	1.000	0.466
22E	1.000	0.617

Extraction Method: Principal Component Analysis.

Total Variance Explained

	Initial Eigenvalues			Extraction Sums of Squared Loadings			Rotation
Component	Total	% of Variance	Cumulative %	Total	% of Variance	Cumulative %	Total
1	3.144	31.441	31.441	3.144	31.441	31.441	2.237
2	1.891	18.905	50.346	1.891	18.905	50.346	1.932
3	1.074	10.744	61.090	1.074	10.744	61.090	1.587
4	1.003	10.031	71.121	1.003	10.031	71.121	1.357
5	0.839	8.386	79.507				
6	0.679	6.793	86.300				
7	0.478	4.783	91.083				
8	0.393	3.932	95.015				
9	0.291	2.906	97.922				
10	0.208	2.078	100.000				

Extraction Method: Principal Component Analysis.

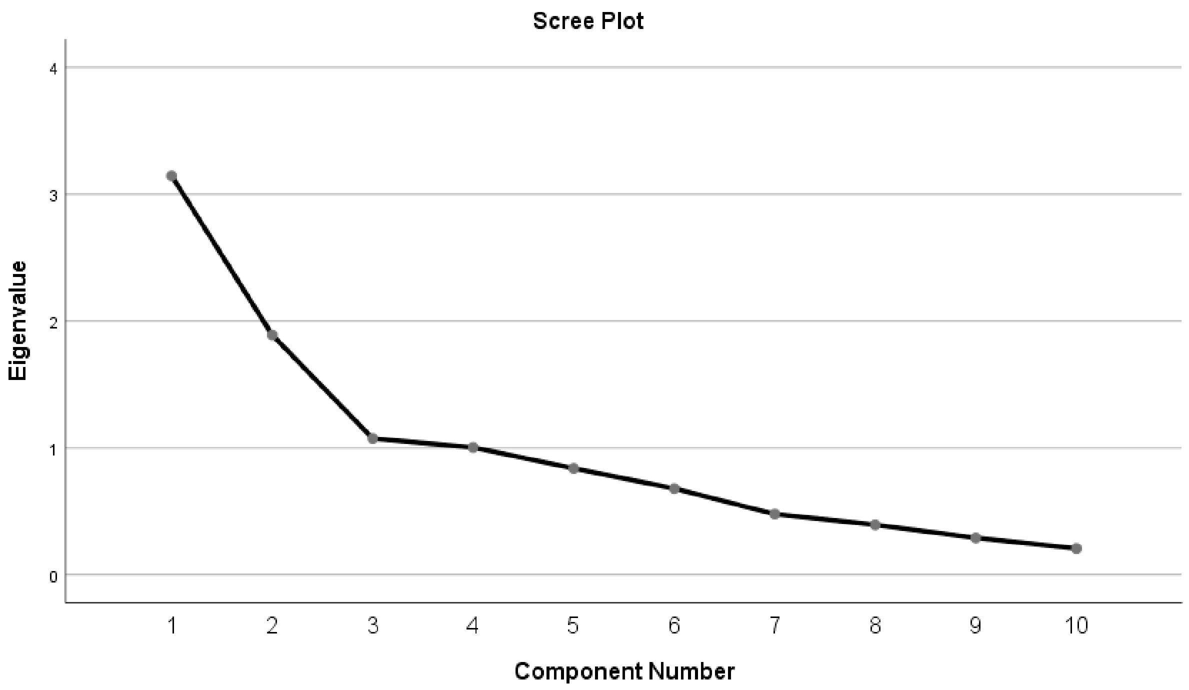

Component Matrix[a]

	Component			
	1	2	3	4
20B	0.319	0.770		
20C	0.541			-0.698
20D	-0.670			0.387
21B	0.766			
21C	0.577	-0.475		
21D	-0.496		-0.719	-0.336
21E	-0.573	0.367	0.537	
22B	0.341	0.837		
22D	0.388	-0.435		0.346

22E	-0.735			

Extraction Method: Principal Component Analysis.

a. 4 components extracted.

Pragmatism & Efficiency vs. human impact score construction

Descriptive Statistics

	Mean	Std. Deviation	Analysis N
8A	2.92	1.100	195
8B	4.24	1.020	195
8C	2.25	1.228	195
8D	2.85	1.409	195
8E	2.74	1.449	195
9A	3.07	1.290	195
9B	4.05	1.159	195
9C	2.75	1.325	195
9D	2.32	1.273	195
9E	2.82	1.413	195
10A	2.67	1.092	195
10B	4.51	0.864	195
10C	2.28	1.216	195
10D	2.76	1.364	195
10E	2.79	1.325	195
11A	2.82	1.225	195
11B	4.31	1.040	195
11C	1.89	1.187	195

11D	3.31	1.157	195
11E	2.68	1.228	195
12A	3.40	1.047	195
12B	3.09	1.148	195
12C	3.15	1.798	195
12D	2.97	1.335	195
12E	2.39	1.433	195
13A	2.10	1.089	195
13B	3.12	1.033	195
13C	4.63	0.765	195
13D	3.04	1.292	195
13E	2.11	1.123	195
14A	3.63	1.134	195
14B	3.89	1.278	195
14C	3.03	1.396	195
14D	1.91	1.165	195
14E	2.53	1.104	195
15A	2.48	1.071	195
15B	3.39	1.293	195
15C	2.47	1.423	195
15D	3.71	1.411	195
15E	2.95	1.415	195
16A	3.06	1.221	195
16B	4.55	0.774	195
16C	3.02	1.266	195
16D	1.78	0.890	195
16E	2.59	1.212	195

17A	2.89	0.957	195
17B	3.43	1.421	195
17C	3.48	1.224	195
17D	2.25	1.400	195
17E	2.96	1.632	195
18A	2.66	1.255	195
18B	3.30	1.283	195
18C	3.96	1.183	195
18D	2.93	1.332	195
18E	2.14	1.335	195
19A	2.57	0.979	195
19B	4.31	0.913	195
19C	4.12	0.838	195
19D	2.04	1.159	195
19E	1.97	1.025	195
20A	3.29	1.041	195
20B	4.35	1.037	195
20C	2.79	1.226	195
20D	2.39	1.290	195
20E	2.16	1.298	195
21A	3.54	1.415	195
21B	3.69	1.373	195
21C	2.73	1.277	195
21D	2.16	0.965	195
21E	2.88	1.431	195
22A	2.38	1.136	195
22B	3.85	1.310	195

22C	2.18	1.263	195
22D	3.39	1.163	195
22E	3.19	1.461	195

Communalities

	Initial	Extraction
8A	1.000	0.716
8B	1.000	0.737
8C	1.000	0.770
8D	1.000	0.792
8E	1.000	0.816
9A	1.000	0.741
9B	1.000	0.719
9C	1.000	0.731
9D	1.000	0.745
9E	1.000	0.746
10A	1.000	0.773
10B	1.000	0.737
10C	1.000	0.770
10D	1.000	0.844
10E	1.000	0.821
11A	1.000	0.735
11B	1.000	0.687
11C	1.000	0.769
11D	1.000	0.702
11E	1.000	0.857

12A	1.000	0.713
12B	1.000	0.689
12C	1.000	0.811
12D	1.000	0.773
12E	1.000	0.694
13A	1.000	0.751
13B	1.000	0.758
13C	1.000	0.746
13D	1.000	0.704
13E	1.000	0.776
14A	1.000	0.750
14B	1.000	0.744
14C	1.000	0.757
14D	1.000	0.756
14E	1.000	0.767
15A	1.000	0.678
15B	1.000	0.772
15C	1.000	0.772
15D	1.000	0.741
15E	1.000	0.678
16A	1.000	0.786
16B	1.000	0.786
16C	1.000	0.804
16D	1.000	0.730
16E	1.000	0.819
17A	1.000	0.748
17B	1.000	0.794

17C	1.000	0.801
17D	1.000	0.742
17E	1.000	0.792
18A	1.000	0.739
18B	1.000	0.737
18C	1.000	0.742
18D	1.000	0.702
18E	1.000	0.774
19A	1.000	0.781
19B	1.000	0.768
19C	1.000	0.757
19D	1.000	0.811
19E	1.000	0.730
20A	1.000	0.691
20B	1.000	0.798
20C	1.000	0.717
20D	1.000	0.784
20E	1.000	0.744
21A	1.000	0.797
21B	1.000	0.782
21C	1.000	0.743
21D	1.000	0.698
21E	1.000	0.752
22A	1.000	0.702
22B	1.000	0.828
22C	1.000	0.798
22D	1.000	0.698

22E	1.000	0.754

Extraction Method: Principal Component Analysis.

Total Variance Explained

	Initial Eigenvalues			Extraction Sums of Squared Loadings		
Component	Total	% of Variance	Cumulative %	Total	% of Variance	Cumulative %
1	11.063	14.750	14.750	11.063	14.750	14.750
2	7.452	9.936	24.686	7.452	9.936	24.686
3	4.413	5.884	30.570	4.413	5.884	30.570
4	3.559	4.746	35.316	3.559	4.746	35.316
5	3.058	4.077	39.393	3.058	4.077	39.393
6	2.799	3.732	43.125	2.799	3.732	43.125
7	2.657	3.543	46.668	2.657	3.543	46.668
8	2.065	2.753	49.421	2.065	2.753	49.421
9	1.953	2.604	52.025	1.953	2.604	52.025
10	1.799	2.398	54.423	1.799	2.398	54.423
11	1.686	2.249	56.671	1.686	2.249	56.671
12	1.559	2.078	58.749	1.559	2.078	58.749
13	1.525	2.033	60.783	1.525	2.033	60.783
14	1.490	1.987	62.769	1.490	1.987	62.769
15	1.446	1.928	64.697	1.446	1.928	64.697
16	1.381	1.842	66.539	1.381	1.842	66.539
17	1.254	1.671	68.210	1.254	1.671	68.210
18	1.222	1.630	69.840	1.222	1.630	69.840
19	1.119	1.492	71.332	1.119	1.492	71.332
20	1.103	1.470	72.803	1.103	1.470	72.803

21	1.050	1.400	74.203	1.050	1.400	74.203
22	1.014	1.352	75.555	1.014	1.352	75.555
23	0.966	1.288	76.842			
24	0.958	1.277	78.120			
25	0.914	1.219	79.339			
26	0.861	1.148	80.487			
27	0.806	1.074	81.561			
28	0.780	1.040	82.601			
29	0.772	1.030	83.630			
30	0.734	0.979	84.610			
31	0.683	0.910	85.520			
32	0.675	0.901	86.420			
33	0.646	0.861	87.281			
34	0.603	0.803	88.085			
35	0.589	0.786	88.871			
36	0.547	0.729	89.600			
37	0.533	0.711	90.311			
38	0.487	0.650	90.961			
39	0.471	0.628	91.589			
40	0.464	0.618	92.207			
41	0.452	0.602	92.809			
42	0.428	0.570	93.380			
43	0.420	0.559	93.939			
44	0.399	0.532	94.471			
45	0.381	0.509	94.980			
46	0.363	0.484	95.464			
47	0.339	0.452	95.917			

48	0.330	0.440	96.356			
49	0.327	0.436	96.792			
50	0.302	0.402	97.194			
51	0.290	0.387	97.581			
52	0.281	0.375	97.956			
53	0.256	0.341	98.297			
54	0.251	0.335	98.632			
55	0.223	0.297	98.929			
56	0.193	0.257	99.187			
57	0.175	0.233	99.420			
58	0.154	0.206	99.626			
59	0.144	0.192	99.818			
60	0.136	0.181	99.999			
61	0.001	0.001	100.000			
62	2.581E-15	3.441E-15	100.000			
63	1.486E-15	1.982E-15	100.000			
64	1.221E-15	1.628E-15	100.000			
65	9.305E-16	1.241E-15	100.000			
66	6.365E-16	8.487E-16	100.000			
67	5.146E-16	6.862E-16	100.000			
68	1.408E-16	1.878E-16	100.000			
69	-9.438E-17	-1.258E-16	100.000			
70	-2.857E-16	-3.810E-16	100.000			
71	-5.521E-16	-7.361E-16	100.000			
72	-8.042E-16	-1.072E-15	100.000			
73	-1.013E-15	-1.351E-15	100.000			
74	-1.564E-15	-2.085E-15	100.000			

75	-1.781E-15	-2.375E-15	100.000			

Extraction Method: Principal Component Analysis.

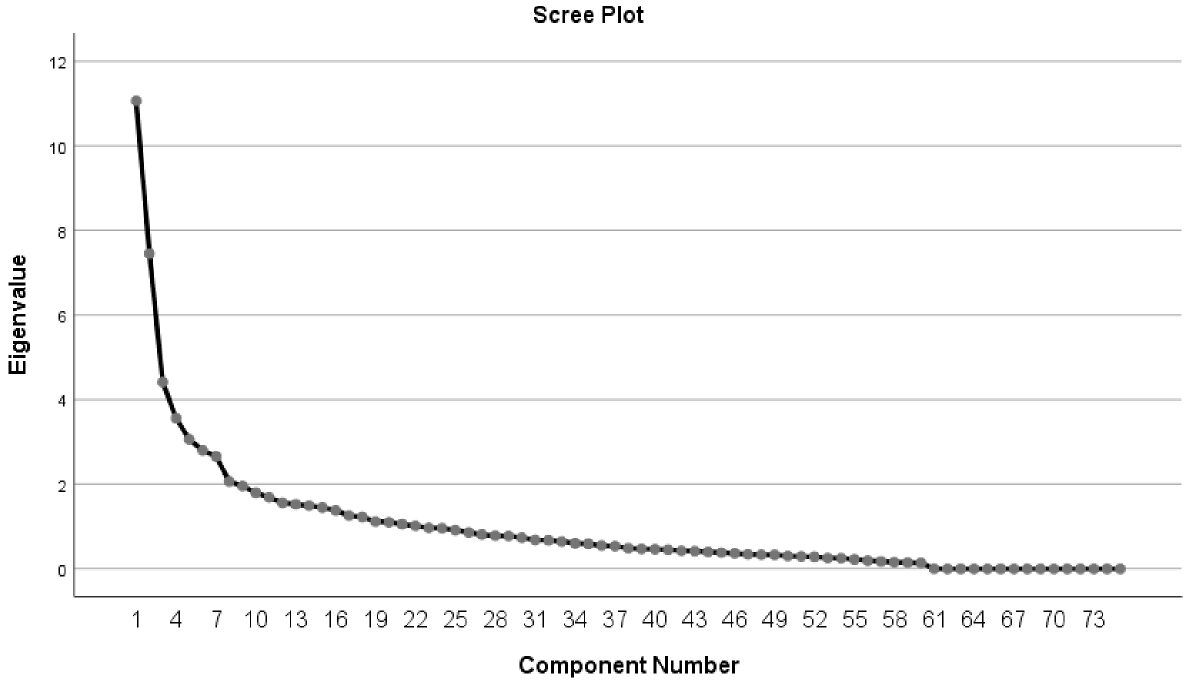

Component Matrix[a]

	Component																					
	1	2	3	4	5	6	7	8	9	10	11	12	13	14	15	16	17	18	19	20	21	22
8A		0.402			-0.321																	
8B		0.411						-0.308						0.321								
8C	-0.471			0.403																		
8D	0.317					0.441			0.323													
8E	0.314	-0.311				-0.492																
9A	-0.315	0.333		0.306																		
9B		0.470					0.417															
9C	0.450																					

9D		-0.361				-0.370	-0.396															
9E	-0.318	-0.326		-0.383																		
10A				0.364						-0.342												
10B							0.434															
10C	-0.527		0.419											-0.340								
10D	0.350				0.330																	
10E					-0.405					-0.379		0.314										
11A		0.311	0.528																			
11B		0.306	-0.376									0.372										
11C	0.310						-0.336						0.353									
11D		-0.311					0.309	-0.378			0.323											
11E								0.470	0.408													
12A	-0.313			0.336		-0.406																
12B	-0.469				0.424			0.313														
12C	0.804																					
12D	0.302							-0.436	0.345													
12E	-0.686																					
13A	-0.401	-0.503		0.395																		
13B				-0.391					-0.383	0.302												
13C		0.408				-0.319	0.357															
13D		0.675																				
13E		-0.342			-0.344							-0.329										
14A	0.387		-0.367	0.521																		
14B	-0.472		0.305			-0.384																
14C	-0.416			-0.490	0.312										-0.301							
14D	0.305					0.347					0.430											
14E	0.352														0.345	-0.352						
15A				0.424	0.423																	
15B				-0.333		0.439																
15C	0.417		0.632																			
15D		0.347	-0.587																			
15E	-0.314		-0.445																			
16A	-0.473	0.368															-0.308					

16B		0.350							0.421													
16C	0.732																					
16D						0.433									-0.360							
16E	-0.340	-0.386					-0.345															
17A			0.370			0.468																
17B		0.727																				
17C	0.646																					
17D		-0.776																				
17E	-0.660																					
18A	-0.391				0.318			-0.366														
18B	-0.649																					
18C	0.324	0.514			0.302																	
18D	0.599																					
18E					-0.530			0.430														
19A				0.473			-0.349															
19B	-0.406		0.485	-0.317																		
19C	0.438	0.301	-0.371									-0.306										
19D	0.351	-0.536																				-0.305
19E	-0.509																					
20A					-0.336																	
20B	0.336	0.527			0.320																	
20C	0.418																					
20D	-0.461									-0.342												
20E		-0.535	-0.364																			
21A	-0.539		0.429																			
21B	0.699																					
21C	0.625																					
21D	-0.433																				0.369	
21E	-0.403	0.473																				
22A		-0.557			0.406																	
22B	0.410	0.554					-0.440															
22C			0.634																			
22D	0.329		-0.344																			

22E	-0.629		-0.343																			

Extraction Method: Principal Component Analysis.

a. 22 components extracted.

Pragmatism final model

Descriptive Statistics

	Mean	Std. Deviation	Analysis N
8C	2.26	1.226	198
8D	2.83	1.407	198
8E	2.74	1.443	198
9C	2.75	1.327	198
10C	2.27	1.212	198
10D	2.74	1.367	198
11C	1.88	1.184	198
12B	3.09	1.139	198
12C	3.15	1.798	198
12D	2.97	1.336	198
12E	2.39	1.441	198
14A	3.62	1.133	198
14B	3.91	1.275	198
14C	3.05	1.390	198
14E	2.53	1.102	198
16A	3.06	1.216	198
16C	3.00	1.275	198
17C	3.47	1.224	198
17E	2.98	1.632	198
18A	2.66	1.252	198

18B	3.31	1.280	198
18D	2.92	1.333	198
19C	4.12	0.834	198
19E	1.97	1.022	198
20C	2.79	1.228	198
20D	2.40	1.294	198
21A	3.55	1.416	198
21B	3.68	1.369	198
21C	2.72	1.274	198
21D	2.16	0.967	198
22E	3.19	1.464	198

KMO and Bartlett's Test

Kaiser-Meyer-Olkin Measure of Sampling Adequacy.		0.760
Bartlett's Test of Sphericity	Approx. Chi-Square	2864.083
	df	465
	Sig.	0.000

Communalities

	Initial	Extraction
8C	1.000	0.670
8D	1.000	0.742
8E	1.000	0.756
9C	1.000	0.444
10C	1.000	0.711

10D	1.000	0.620
11C	1.000	0.642
12B	1.000	0.707
12C	1.000	0.769
12D	1.000	0.776
12E	1.000	0.667
14A	1.000	0.736
14B	1.000	0.674
14C	1.000	0.658
14E	1.000	0.557
16A	1.000	0.679
16C	1.000	0.758
17C	1.000	0.703
17E	1.000	0.717
18A	1.000	0.580
18B	1.000	0.559
18D	1.000	0.649
19C	1.000	0.678
19E	1.000	0.668
20C	1.000	0.710
20D	1.000	0.606
21A	1.000	0.629
21B	1.000	0.672
21C	1.000	0.554
21D	1.000	0.607
22E	1.000	0.671

Extraction Method: Principal Component Analysis.

Total Variance Explained

	Initial Eigenvalues			Extraction Sums of Squared Loadings			Rotation Sums of Squared Loadings		
Component	Total	% of Variance	Cumulative %	Total	% of Variance	Cumulative %	Total	% of Variance	Cumulative %
1	8.638	27.866	27.866	8.638	27.866	27.866	3.679	11.867	11.867
2	2.240	7.227	35.093	2.240	7.227	35.093	2.801	9.035	20.902
3	1.923	6.204	41.297	1.923	6.204	41.297	2.695	8.694	29.597
4	1.638	5.284	46.581	1.638	5.284	46.581	2.505	8.079	37.676
5	1.487	4.796	51.377	1.487	4.796	51.377	1.993	6.428	44.104
6	1.252	4.039	55.416	1.252	4.039	55.416	1.982	6.394	50.497
7	1.188	3.832	59.247	1.188	3.832	59.247	1.739	5.609	56.106
8	1.118	3.605	62.853	1.118	3.605	62.853	1.676	5.406	61.513
9	1.085	3.499	66.352	1.085	3.499	66.352	1.500	4.839	66.352
10	0.978	3.156	69.508						
11	0.926	2.988	72.495						
12	0.816	2.631	75.127						
13	0.786	2.534	77.661						
14	0.758	2.444	80.104						
15	0.662	2.135	82.239						
16	0.584	1.885	84.125						
17	0.573	1.849	85.974						
18	0.530	1.710	87.684						
19	0.488	1.575	89.259						

20	0.457	1.475	90.733						
21	0.436	1.406	92.139						
22	0.391	1.263	93.401						
23	0.381	1.229	94.631						
24	0.349	1.125	95.756						
25	0.278	0.897	96.652						
26	0.244	0.788	97.440						
27	0.222	0.717	98.156						
28	0.209	0.676	98.832						
29	0.172	0.555	99.388						
30	0.112	0.361	99.749						
31	0.078	0.251	100.000						

Extraction Method: Principal Component Analysis.

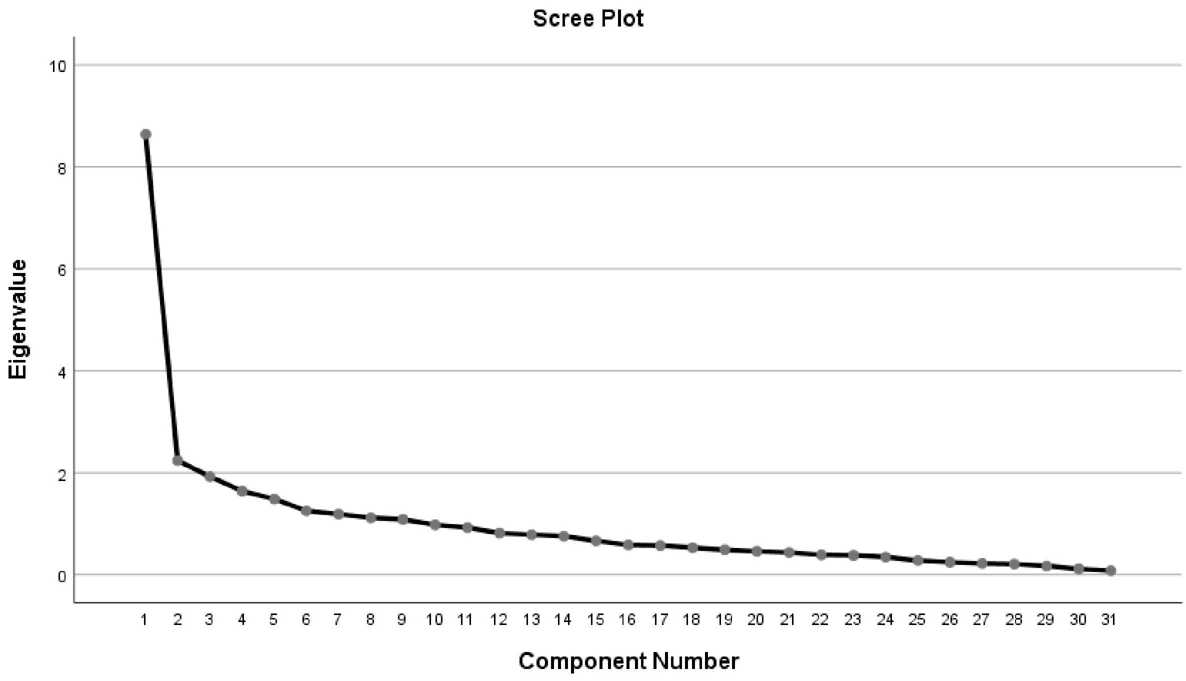

Component Matrix[a]

	Component								
	1	2	3	4	5	6	7	8	9
8C	0.481			0.564					
8D	-0.329	-0.479				-0.436			
8E	-0.306	0.521				0.444			
9C	-0.452			0.332					
10C	0.559			0.401					
10D	-0.385		0.390				-0.494		
11C	-0.307						0.541	-0.400	
12B	0.488	0.327			0.515				
12C	-0.792		-0.308						
12D	-0.310	-0.327	0.507		-0.311			0.361	
12E	0.697								
14A	-0.439	-0.578		0.350					
14B	0.499	0.431			-0.488				
14C	0.443	0.319	0.340	-0.363					
14E	-0.370		-0.557						
16A	0.467		-0.349			0.373			
16C	-0.717	0.360							
17C	-0.633				0.415				
17E	0.644				-0.345				
18A	0.347		0.395			0.375			
18B	0.653								
18D	-0.614							0.303	

19C	-0.442			-0.314					0.461
19E	0.501								-0.571
20C	-0.430			0.464					0.363
20D	0.504			-0.304	0.366				
21A	0.581	0.427							
21B	-0.708								
21C	-0.670								
21D	0.457				0.308	-0.415			
22E	0.610	-0.381	-0.303						

Extraction Method: Principal Component Analysis.

a. 9 components extracted.

Efficency vs. human impact final model

Descriptive Statistics

	Mean	Std. Deviation	Analysis N
8A	2.93	1.102	200
8B	4.26	1.014	200
9B	4.06	1.148	200
9D	2.32	1.270	200
9E	2.83	1.412	200
13A	2.10	1.084	200
13C	4.61	0.789	200
13D	3.04	1.291	200
13E	2.12	1.137	200
16B	4.56	0.768	200

16E	2.61	1.215	200
17B	3.42	1.415	200
17D	2.24	1.397	200
18C	3.95	1.189	200
19D	2.03	1.151	200
20B	4.35	1.036	200
20E	2.17	1.304	200
21E	2.90	1.426	200
22A	2.38	1.127	200
22B	3.85	1.314	200

KMO and Bartlett's Test

Kaiser-Meyer-Olkin Measure of Sampling Adequacy.		0.748
Bartlett's Test of Sphericity	Approx. Chi-Square	1386.640
	df	190
	Sig.	0.000

Communalities

	Initial	Extraction
8A	1.000	0.554
8B	1.000	0.264
9B	1.000	0.577
9D	1.000	0.764
9E	1.000	0.779
13A	1.000	0.697

13C	1.000	0.670
13D	1.000	0.658
13E	1.000	0.805
16B	1.000	0.507
16E	1.000	0.666
17B	1.000	0.595
17D	1.000	0.690
18C	1.000	0.654
19D	1.000	0.457
20B	1.000	0.712
20E	1.000	0.639
21E	1.000	0.652
22A	1.000	0.542
22B	1.000	0.771

Extraction Method: Principal Component Analysis.

Total Variance Explained

	Initial Eigenvalues			Extraction Sums of Squared Loadings			Rotation Sums of Squared Loadings		
Component	Total	% of Variance	Cumulative %	Total	% of Variance	Cumulative %	Total	% of Variance	Cumulative %
1	5.475	27.374	27.374	5.475	27.374	27.374	3.004	15.022	15.022
2	2.035	10.173	37.547	2.035	10.173	37.547	2.411	12.057	27.079
3	1.468	7.342	44.889	1.468	7.342	44.889	2.367	11.836	38.915
4	1.328	6.641	51.531	1.328	6.641	51.531	1.752	8.760	47.675
5	1.212	6.060	57.590	1.212	6.060	57.590	1.685	8.423	56.098

6	1.136	5.678	63.268	1.136	5.678	63.268	1.434	7.170	63.268
7	0.938	4.688	67.956						
8	0.881	4.406	72.363						
9	0.723	3.616	75.979						
10	0.706	3.530	79.509						
11	0.673	3.366	82.874						
12	0.574	2.872	85.746						
13	0.569	2.845	88.591						
14	0.474	2.370	90.961						
15	0.417	2.085	93.046						
16	0.412	2.058	95.104						
17	0.306	1.530	96.635						
18	0.278	1.390	98.024						
19	0.239	1.195	99.220						
20	0.156	0.780	100.000						

Extraction Method: Principal Component Analysis.

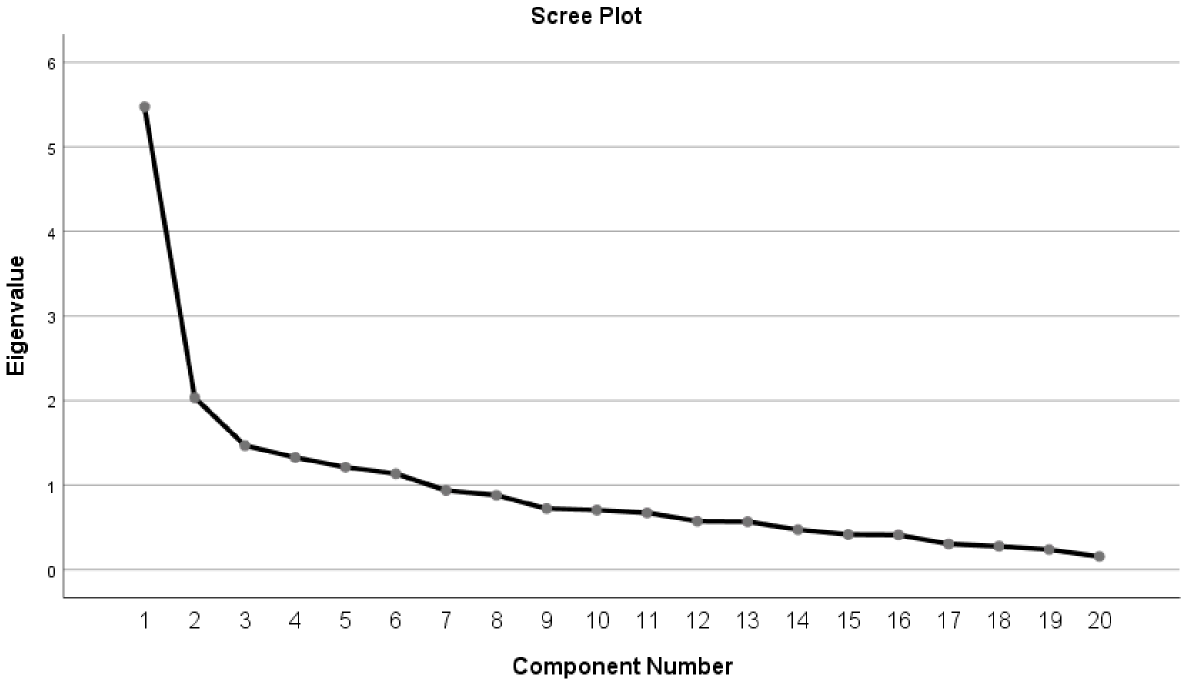

Component Matrix[a]

	Component					
	1	2	3	4	5	6
8A	0.379	-0.335		0.412		
8B	0.338					
9B	0.526		0.447			
9D	-0.369	0.417	-0.520			0.384
9E	-0.365		0.446		0.569	
13A	-0.589	-0.412		-0.301		
13C	0.392		0.424			0.550
13D	0.660		-0.306			
13E	-0.321	0.519		0.486		-0.384
16B	0.439		0.364			
16E	-0.504			0.491	0.384	

17B	0.689					
17D	-0.780					
18C	0.553				0.479	
19D	-0.439	0.344		-0.355		
20B	0.619	0.462				
20E	-0.563					0.432
21E	0.372	-0.460			0.448	
22A	-0.598			-0.331		
22B	0.638	0.480				

Extraction Method: Principal Component Analysis.

a. 6 components extracted.
